# Defining the Ovarian Cancer Precancerous Landscape through Modeling Fallopian Tube Epithelium Reprogramming Driven by Extracellular Vesicles

**DOI:** 10.1158/2767-9764.CRC-25-0064

**Published:** 2025-08-04

**Authors:** Jared Sipes, Didi Zha, Sagar Rayamajhi, Leonidas E. Bantis, Rashna Madan, Amrita Mitra, Rajni V. Puri, Mohammod Mahmudur Rahman, Foyez Ahmmed, Harsh B. Pathak, Angela Russo, Mihaela Sardiu, Brett C. Isenberg, Brian P. Cain, Jonathan Coppeta, Pamoda M. Galhenage, Shailja Pathania, Shannon MacLaughlan David, Joanna E. Burdette, Andrew K. Godwin

**Affiliations:** 1 Department of Pathology and Laboratory Medicine, University of Kansas Medical Center, Kansas City, Kansas.; 2 Bioengineering Program, The University of Kansas, Lawrence, Kansas.; 3 Department of Pharmaceutical Sciences, College of Pharmacy, University of Illinois at Chicago, Chicago, Illinois.; 4 Department of Biostatistics and Data Science, University of Kansas Medical Center, Kansas City, Kansas.; 5 The University of Kansas Cancer Center, University of Kansas Medical Center, Kansas City, Kansas.; 6 The Kansas Institute for Precision Medicine, University of Kansas Medical Center, Kansas City, Kansas.; 7 Department of Statistics, Comilla University, Cumilla, Bangladesh.; 8 Charles Stark Draper Laboratory, Cambridge, Massachusetts.; 9 Center for Personalized Cancer Therapy, University of Massachusetts, Boston, Massachusetts.; 10 Department of Biology, University of Massachusetts Boston, Boston, Massachusetts.; 11 Translational Oncology Program, University of Illinois Cancer Center, University of Illinois at Chicago, Chicago, Illinois.

## Abstract

Serous tubal intraepithelial carcinomas (lesions) in the human fallopian tube epithelium (hFTE) are theorized to give rise to high-grade serous ovarian cancers. Small extracellular vesicles (sEV) are known to mediate key signaling in both normal and cancerous tissues, but few *ex vivo* systems exist for studying the impact of sEV on hFTE tissue. In this study, we present a microfluidic tissue culture platform with combined spatial transcriptomic and proteomic readouts that allows us to profile dual responses in tissue exposed to sEV “messages”—capturing both short-term transcriptomic shifts in the tissue and long-term changes in protein cargo of secreted EVs (the “reply”). Using spatial transcriptomics, we show that the short-term 1-day exposure to ovarian cancer–derived sEVs alters expression of 68 transcripts in secretory cells, the progenitor of high-grade serous ovarian cancer, notably upregulating immune-related mRNA, including CXCL family chemokines, *VCAM1*, and pro-inflammatory mediators (*NFKB1*, *IL1B*, and *IFNA7/17*). Additionally, we observed that the long-term 14-day exposure to sEVs alters the expression of seven transcripts and 25 EV cargo proteins of fallopian tube–derived EVs (“secondary release EVs”) following stimulus from cancer EVs. Together, tissue transcriptomics and tissue-derived EV proteomics indicate that ovarian cancer–derived sEVs rewire target cell signaling to modify the tubal immune landscape. This study provides insights into the early molecular changes associated with the pathogenesis of ovarian cancer in its tissue of origin, providing a platform to study EV–tissue interactions and identify how sEVs drive cell signaling reprogramming in hFTE.

**Significance:**

We model the fallopian tube preneoplastic landscape using a microfluidic platform to study EV-induced stress and show that cancer EVs promote immune signaling changes representing the earliest stages of ovarian cancer pathogenesis.

## Introduction

The fallopian tube is vital to female reproductive health, playing a central role in fertility and gynecologic pathology. The fallopian tube is not simply a conduit between the uterus and ovaries but a dynamic organ that regulates early fertilization and embryonic development (1). Growing evidence points to the human fallopian tube epithelium (hFTE) as the site of origin of high-grade serous ovarian cancer (HGSOC; ref. 2), the most lethal and common subtype of ovarian cancer. Precursor lesions of HGSOC, such as secretory cell expansions, secretory cell outgrowth, and p53 signatures, exist throughout the fallopian tube, and serous tubal intraepithelial carcinoma (STIC) lesions are enriched in the fimbriated end of the fallopian tube (3–5). In addition, epithelial ovarian cancer risk correlates with lifetime ovulations: Earlier menarche and late onset of menopause increase risk, whereas pregnancy, breastfeeding, and oral contraceptives, which reduce ovulation, are protective (6). Based on these findings, it is hypothesized that ovarian secretions (e.g., estrogen) or follicular fluid components (e.g., reactive oxygen species) contribute to ovarian cancer either directly via mutagenesis or indirectly by promoting metastasis to the ovary (7). Although small molecules and hormonal components involved in cross-talk between ovarian cancer, the fallopian tube, and the ovaries have been studied, more complex signaling mechanisms, such as those mediated by extracellular vesicles (EV), may also be involved in early tumorigenesis and require further exploration.

Recent research highlights the role of EVs in both normal tubal function (8) and ovarian cancer development (9). EVs are lipid membrane–bound particles secreted by cells which facilitate intercellular signaling by transferring biologically active cargo—such as miRNA, protein, and metabolites—to recipient cells (10). Although cells produce a wide array of EVs, most studies focus on small EVs (sEV). sEVs are approximately 50 to 200 nm in diameter and include both “ectosomes,” which bud directly from the cell membrane, and “exosomes,” which are produced in a multivesicular body and released in mass. Tumor cells secrete more sEVs than healthy cells with altered cargo that facilitates tumorigenesis (11), and cancer-derived sEVs have been shown to contribute to nearly every stage of cancer progression from initiation to metastasis and chemoresistance (12). Additionally, EVs provide a “snapshot” of selectively packaged molecules from their cells of origin, making them promising carriers of biomarkers for detecting early cancer, tracking tumor progression, and monitoring therapeutic response (12).

EV signaling plays an important role in the mammalian oviduct, in which oviductal sEVs enhance oocyte maturation, increase fertilization rate, and prevent polyspermy (8). Ovarian cancer progression is also mediated by EVs, which aid chemoresistance, metastasis, and immune evasion (13, 14). However, it is not known whether sEVs released by cancer cells in the fallopian tube and ovarian microenvironment alter signaling in adjacent healthy FTE cells. Adding complexity, EV signaling is bidirectional: recipient cells can release their own EVs in response, creating biochemical “replies” that may further contribute to disease progression. These “secondary release sEVs” could be more abundant than cancer-derived sEVs and present a promising, untapped source of early cancer biomarkers (12).

Investigating these dynamics is hindered by lack of suitable model systems to explore complex EV–tissue interactions. Conventional fallopian tube cell cultures grown on two-dimensional monolayers lose polarity and viability unless immortalized, which requires hTERT overexpression and disruption of tumor-suppressor pathways (15). Additionally, immortalized lines retain secretory cell features but lose ciliated cell populations, limiting their utility for modeling EV–tissue interactions.

To overcome these limitations, we developed a microfluidic tissue culture platform with a dynamic air–liquid interface (ALI) to sustain the morphology and function of hFTE. ALI culture, widely adopted in airway epithelial cell models, maintains differentiated populations more effectively than submerged cultures (16). We previously demonstrated that hFTE tissues cultured on an ALI platform maintained cilia beating and distinct ciliated and secretory cell populations after 14 days (17). Building on this, we optimized the method using the PREDICT Multi-Organ System (PREDICT-MOS) for long-term culture of FT explants and profiled normal-tissue EV production (18).

In the current study, using the PREDICT-MOS system, we cultured primary hFTE tissue explants and exposed them to EVs derived from HGSOC cells (OVCAR3) and non-tumorigenic fallopian tube secretory cells (FT240) over short- (1-day) and long-term (14-day) periods. Using HGSOC-derived EVs as an extreme source of insult, in these proof-of-principle experiments, we aim to determine whether ovarian cancer–derived EVs can induce pro-oncogenic signaling in the tubal epithelia that can sustain and enhance malignant progression. To investigate the effects of EV exposure, we used spatial transcriptomic profiling to characterize tissue mRNA transcript changes and mass spectrometry proteomics to characterize changes in tissue-derived sEV protein cargo. These approaches allowed us to assess changes in the FTE and exo-proteome induced by EV insult.

We observed that sEVs added to the donor well of the PREDICT microfluidic were taken up by hFTE explants, leading to transcriptomic changes at both 1- and 14-day time points. In total, the study included 12 unique fallopian tube samples in the short-term exposure dataset, 10 samples in the long-term dataset, and five hFTE-derived sEV samples for proteomic analysis.

Transcriptomic analysis revealed 50 differentially expressed genes (DEG) induced solely by OVCAR3 sEVs (but not FT240-derived sEVs) in secretory cells and 38 DEGs in ciliated cells following short-term exposure. Many of the DEGs were cytokines (*CXCL1* and *CXCL5*), immune related (*NFKB1* and *IL1B*), or cell adhesion related (*VCAM1*), highlighting alterations in immune signaling and the epithelial–immune microenvironment. Gene ontology (GO) analysis confirmed activation of pathways related to immune cell migration and response, suggesting substantial remodeling of the local immune landscape by ovarian cancer EVs.

This new EV–tissue interaction model provides insights into the interplay between ovarian cancer–derived EVs and FTE cells, advancing our understanding of early-stage tumorigenesis and offering potential avenues for biomarker discovery.

## Materials and Methods

### Human fallopian tube tissue collection

The studies were approved by the appropriate institutional research ethics committees at University of Illinois Chicago (UIC) and Kansas University Medical Center (KUMC) and were performed in accordance with the ethical standards, as laid down in the 1964 Declaration of Helsinki and its later amendments or comparable ethical standards. The use of human samples was approved by the Institutional Review Board (IRB) under the Human Tissue Bank Protocol at UIC (IRB #2017-0574) or the Biospecimen Repository Core Facility (HSC #5929) at KUMC. De-identified fallopian tube tissues were obtained from women who provided informed consent.

### IRB statement

The studies have been approved by the appropriate institutional research ethics committee and have been performed in accordance with the ethical standards, as laid down in the 1964 Declaration of Helsinki and its later amendments or comparable ethical standards. The use of human samples was approved by the IRB under the Human Tissue Bank Protocol at UIC (IRB #2017-0574) or the Biospecimen Repository Core Facility (HSC #5929) at the KUMC. De-identified fallopian tube tissues were obtained from women who provided informed consent.

### Informed consent statement

All participants provided written informed consent prior to participating in the study, signifying their understanding of the research procedures, potential risks and benefits, and their right to withdraw at any time without penalty. Once the patient provided written informed consent in accordance with the respective IRB protocols, specimens were procured at the time of clinically indicated surgery and fallopian tube tissue not required for diagnostic purposes was placed directly into medium. All samples were de-identified to the user prior to transport to the laboratory for processing.

### Purification and characterization of EVs used for tissue treatment

Conditioned medium was collected from OVCAR3 (HGSOC model, RRID: CVCL_0465; ref. 19) or FT240 (immortalized non-tumorigenic fallopian tube, RRID: CVCL_UH60) cell lines (15). Cell line–derived EVs were purified using differential ultracentrifugation (full procedure in Supplementary Methods). Cell lines were tested for *Mycoplasma* contamination using the Tribioscience Mycoplasma Species qPCR Kit (cat. #TBS42030-100).

EV particle count and size were estimated using the NanoSight LM10 Nanoparticle Tracking Analysis (NTA) system (v2.3 Nanosight Ltd.). All readings used identical settings to ensure sample comparability (20°C temperature, cameral level 12, and detection threshold 4). The Bradford assay (20) was used to estimate protein concentration in EV samples.

### Western Assay validation of EV markers

EV markers (CD9, CD81, and FLOT1) were validated by Simple Western assay using ProteinSimple's Wes instrument(Wes, ProteinSimple, catalog # 004-600), which separates proteins in a capillary, followed by immunodetection. WES was run following the manufacturer’s protocol using the 12 to 230 kDa separation module (ProteinSimple, #SM-W004). The assay used cell and EV lysates at 0.6 and 0.4 mg/mL concentrations, respectively. We used primary antibodies against CD9 (Cell Signaling Technology, cat. #13174, RRID: AB_2798139, 1:25 dilution), CD81 (Cell Signaling Technology, cat. #56039, RRID: AB_2924772, 1:25 dilution), and FLOT1 (Santa Cruz Biotechnology, cat. #sc-74566, RRID: AB_2106563, 1:25). A secondary anti-rabbit or anti-mouse module was used for detection. Blot images were captured using Compass software v6.0.0. (ProteinSimple) with a high dynamic range of 4.0. PBS control was used for each detection antibody as a negative control for nonspecific signals.

### Transmission electron microscopy validation of EVs

We have previously described the characterization of sEVs using transmission electron microscopy (see Supplementary Methods for the procedure; ref. 18). A 1% uranyl acetate–negative stain was performed for EV visualization using a JEOL JEM-1400 electron microscope.

### Proteomics characterization of cell line–derived sEVs used for tissue treatment

The proteomic profile of sEVs derived from OVCAR3 and FT240 cell lines was carried out using label-free LC/MS-MS mass spectrometry by the IDeA National Resource for Quantitative Proteomics at the University of Arkansas for Medical Sciences using an Orbitrap Eclipse Tribrid mass spectrometer (Thermo Fisher Scientific). Protein identification was accepted if the FDR was <1% and there were at least two identified peptides. Protein probabilities were assigned by the Protein Prophet algorithm (21). A list of detected proteins is provided in Supplementary Data File S1.

### Primary human fallopian tube dissection, culture, and exposure to sEVs on the PREDICT-MOS

The human fallopian tube tissue was stored in dissection medium (DMEM/Nutrient Mixture F-12 medium, 1:1) with 10% FBS and dissected within 24 hours of arrival. The epithelium was isolated from the muscular layer and part of the underlying stroma using blunt dissection and divided into 2 × 2 mm sections as described previously (22), noting that the stroma immediately underlying the epithelium is preserved. hFTE sections were transferred to 0.4-μm-pore Millicell inserts with growth medium (minimum essential medium with 0.3% BSA, 1% penicillin/streptomycin, and insulin, transferrin, and selenium medium). Cilia beating was confirmed under a bright-field microscope before being transferred to a PREDICT-MOS tissue plate. hFTE sections were treated with PBS, FT240-derived sEVs, or OVCAR3-derived sEVs for 24 hours or 14 days on PREDICT-MOS. For the 14-day treatment, the hFTE-conditioned medium was collected daily, and fresh growth medium with treatment was replenished daily. The tissue was treated with 1.5 × 10^9^ sEVs from FT240 or OVCAR3 cells every 24 hours.

### Validation of EV uptake in the FTE

We recently described the production of a new cell line derived from the FT240 cell line, which simultaneously expresses CD9-mCherry, CD63-mCherry, and CD81-mCherry constructs (23). The CD9-CD63-CD81 FT240 cell lines produce EVs with high expression of mCherry conjugated to these common EV surface proteins, allowing easy tracking of EV uptake in tissue culture. The parental FT240 cell line was kindly provided by Dr. Ronny Drapkin (University of Pennsylvania). Conditioned medium was collected from the FT240 CD9/CD63/CD8-mCherry cell line, and sEVs were purified via differential ultracentrifugation. FT240-mCherry EV uptake was tested in the parental (no-mCherry) FT240 cell line. FT240 (no-mCherry) cells were cultured in eight-well plates, then treated with either mCherry-FT240 EVs (2 μg of total EV protein), no-mCherry FT240 EVs (2 μg total EV protein), or PBS, and then fixed and stained with DAPI for imaging. DAPI and mCherry fluorescence were then measured using confocal fluorescent imaging. EV uptake was quantified in terms of corrected total cell fluorescence (CTCF). CTCF represents the total fluorescence of the cell quantified based on integrated density from ImageJ (red channel) with background subtraction. CTCF was calculated by using the following equation: CTCF = integrated density of cell − (area of selected cell x mean fluorescence of background reading) as previously described (23).

FTE was dissected and cultured on the Millicell Cell Culture Insert (0.4 μm), followed by treatment with 1.5 × 10^10^ FT240 mCherrys EVs for 24 hours. Tissue uptake of mCherry was validated via IHC.

### GeoMx digital spatial profiling slide preparation for hFTE tissue

We have previously described the characterization of microfluidic-cultured hFTE (18) using the GeoMx Digital Spatial Profiling (DSP) platform (NanoString). The full procedure is available in NanoString MAN-10130 (RNA Manual Slide Prep Reagents). Briefly, tissue slides were rehydrated, followed by antigen retrieval using 1×Tris-EDTA solution in a steamer for 20 minutes. A library of RNA probes for 1,814 cancer-related genes was hybridized to the tissue overnight [Cancer Transcriptome Atlas (CTA)]. Following hybridization, fluorescent antibodies against FOXJ1 (ciliated cell marker; Thermo Fisher Scientific, cat. #14-9965-82, RRID: AB_1548835) and PAX8 (secretory cell marker; Proteintech, cat. #10336-1-AP, RRID: AB_2236705) were used to label epithelial cell types in the fallopian tube and SYTO13 was used to stain the nuclei. After scanning on the GeoMx DSP platform, regions of interest (ROI) were manually drawn using the polygon tool to select the epithelium. Automated on-device segmentation was used to further separate the epithelium into ciliated and secretory segments based on FOXJ1 and PAX8 staining. Hybridized probes were collected from FOXJ1-positive and PAX8-positive segments in selected ROIs. Collected probes were quantified using next-generation paired-end (27 × 2 cycles) sequencing on an Illumina NextSeq 550 instrument using a high-output 75-cycle flow cell.

### Spatial transcriptomics quality control, normalization, and data analysis

Quality control and data analyses follow the vignette “Analyzing GeoMx-NGS RNA Expression Data with GeoMxTools” provided by NanoString and use the GeoMxWorkflows (24), NanoStringNCTools, and GeoMxTools packages. Low-quality segments with any of the following characteristics were excluded: <1,000 reads, <80% trimmed, <80% stitched, <75% aligned, <50% saturated, <1 negative probe count, >3,000 no template control count, and <20 nuclei.

In addition, segments with <10% of the panel genes detected were removed from the dataset, along with transcripts detected in <10% of the segments. Normalization was performed using Q3 normalization. All analyses were performed using R version 4.3.1.

### Statistical analyses

#### Differential gene expression analysis between segments

We used a two-sided *t* test to compare transcripts between ciliated and secretory segments. The corresponding *P* value and log fold change (FC) for each marker are reported in Supplementary Data File S2.

#### Differential gene expression analysis between treatment conditions

Transcript expression was compared for all treatment conditions (OVCAR3 vs. FT240, OVCAR3 vs. PBS, and FT240 vs. PBS) in both secretory and ciliated segments using a two-sided unpaired *t* test. For *P* values and log FCs for each comparison, see Supplementary Data File S3.

#### GO analysis, Kyoto Encyclopedia of Genes and Genomes, and Reactome pathway analysis

GO analysis was performed using sets of differentially expressed transcripts and the online resource ShinyGO (v.0.82), with the list of genes present in the CTA panel used as background (25). Cases in which the CTA panel identified multiple genes rather than a single unique transcript were excluded (http://bioinformatics.sdstate.edu/go/). We used ConsensusPathDB to investigate the biological pathways associated with proteins that were either upregulated or downregulated in our dataset. This resource helps identify subnetworks and biological functions related to specific lists of molecules. Using this approach, we identified the top enriched biological pathways from both the Reactome and Kyoto Encyclopedia of Genes and Genomes databases using overrepresentation analysis (using the whole genome/proteome, without background genes or proteins). We defined a pathway as enriched if FDR <0.05.

#### Analysis of external datasets

Protein cargo similarity of HGSOC-derived EVs was analyzed using data from a previously published study (26). Comparison of EV-induced gene expression with transcript expression in HGSOC precursor lesions was performed using data from Gene Expression Omnibus at GSE281193, following the normalization and differential gene expression analysis described by the authors (27).

### IHC staining of fallopian tube slides

IHC was used to stain the tissue slides for markers of interest. Primary antibodies against CCL2 (Novus, cat. #NBP1-07035SS, RRID: AB_1625611), VCAM1 (Thermo Fisher Scientific, cat. #MA5-31965, RRID: AB_2809259), FLNA (Proteintech, cat. #67133-1-Ig, RRID: AB_2882432), TPI1 (Proteintech, cat. #10713-1-AP, RRID: AB_2207716), and TXNIP (Thermo Fisher Scientific, cat. #40-3700, RRID: AB_2533462) were incubated overnight at 4°C, followed by detection using a horseradish peroxidase–linked secondary antibody and DAB substrate (the full procedure is provided in the Supplementary Methods).

Immunofluorescence (IF) staining for markers of DNA damage (53BP1) was performed according to a previously described procedure (28), using a primary antibody against 53BP1 (Thermo Fisher Scientific, cat. #A300-272A, RRID: AB_185520 1:200). For PAX2 IF staining, the primary antibody against PAX2 (Abcam, cat. #ab79389, RRID: AB_1603338) was detected using an anti-rabbit secondary conjugated to Alexa 488 (Thermo Fisher Scientific, cat. #A-11034, RRID: AB_2576217). See Supplementary Methods for full IF procedures.

For quantification, six to 10 ROIs were selected in the epithelial region of the tissue. The corrected total fluorescence (CTF) of the ROI was calculated based on the total integrated density of the ROI minus the integrated density of the background. CTF was calculated by using the following equation: CTF = integrated density of ROI − (area of selected ROI x mean fluorescence of background reading).

### Proteomics characterization of hFTE-derived sEVs

Conditioned medium was collected from OVCAR3 sEV, FT240 sEV, and PBS-treated hFTE cultured tissue explants over a 2-week period. We collected 1.5 mL of conditioned medium daily for a total of 21 mL at the end of collection. EVs were purified via ultracentrifugation, followed by protein quantification using the Bradford assay.

Because of anticipated differences in yield, only five tissues yielded sufficient protein content under all three treatment conditions for mass spectrometry analysis for a total of 15 EV samples (five OVCAR3 treatment condition, five FT240 treatment condition, and five PBS treatment condition). Mass spectrophotometry analysis was once again performed by the IDeA National Resource for Quantitative Proteomics using the Orbitrap Exploris 480 mass spectrometer (the full procedure is provided in the Supplementary Methods).

#### Differential protein expression analysis of EV proteins

Proteomic analysis detected 1,734 proteins across 15 samples. Of these, 631 had <3 observations in at least one treatment group and were excluded. Statistical analysis was performed on the remaining 1,103, and the unpaired *t* test was used to identify statistically significant differences between the treatment groups. The results are shown in Supplementary Data File S4.

### Data availability

Data are available upon request from the corresponding author. Additional data can be found in the Supplementary Data Files S1–S4. Processed data files and code used for quality control, normalization, and graphing are available at the Github repository archived in Zenodo at DOI 10.5281/zenodo.15808861. Complete raw data files are deposited in Gene Expression Omnibus at GSE300490. The mass spectrometry proteomics data have been deposited to the ProteomeXchange Consortium via the PRIDE partner repository with the dataset identifier PXD066017.

## Results

### Dynamic organ-on-chip system permits long-term study of EV–tissue interactions

Primary hFTE cells lose polarity and cellular differentiation in two-dimensional culture (29); however, three-dimensional microfluidic culture with perfusion and an ALI supports the maintenance of diverse cellular populations in primary fallopian tube tissue, retaining viability for up to 28 days (17). We have established a multi-organ reproductive tract microfluidic model (17) and optimized it for culture of human fallopian tubes and murine ovaries (7, 17, 18, 30). Conditioned medium from cultured hFTE explants can be collected, allowing for purification and characterization of hFTE tissue–derived EVs and their proteome (18). Here, we extended the PREDICT platform to study interactions between ovarian cancer–derived sEVs and the hFTE.

**Figure 1:**
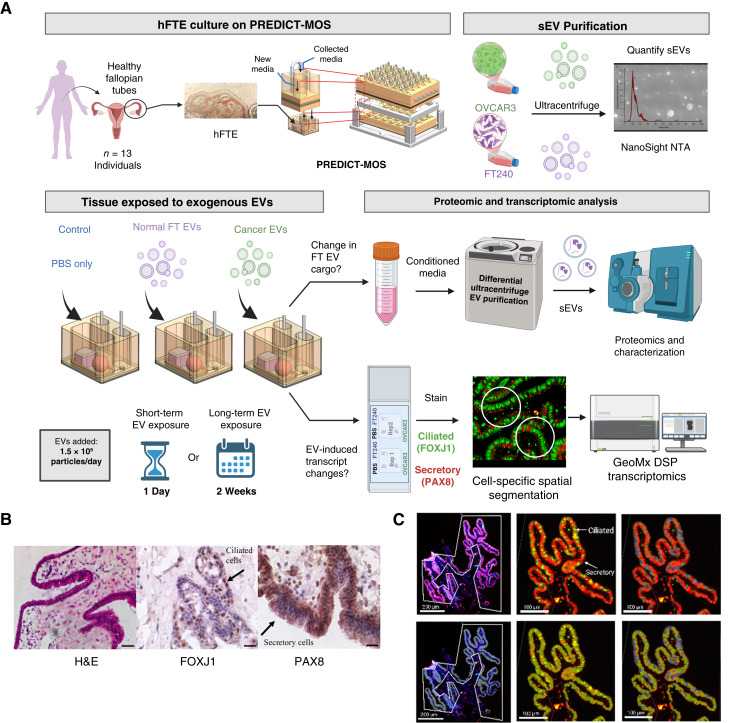
Experimental design and characterization of PREDICT-MOS–cultured FTE. **A,** Diagram showing culture of hFTE and experimental setup including culture on platform (time course and exposures), EV purification, and proteomic and spatial transcriptomic analysis. **B,** IHC staining for secretory cell marker PAX8 and ciliated cell marker FOXJ1 after FTE was cultured on PREDICT-MOS for 14 days. H&E, hematoxylin and eosin. **C,** Example of ROI selection and segmentation on GeoMx DSP. Top left, ROI (white lines) drawn around FTE stained for DAPI, FOXJ1, and PAX8; top middle, staining of ciliated cells (green) and secretory cells (red); top right, automatic identification of secretory cells (blue mask); bottom left, fully segmented ROI; bottom middle, automatic identification of secretory cells (green mask); and bottom left, fully segmented region (green mask = secretory cells and blue mask = ciliated cells).

To study the impact of sEVs from HGSOC (OVCAR3) cells or immortalized fallopian tube (FT240) cells, we cultured primary human tissue on the PREDICT-MOS system and studied changes in the spatial transcriptome and the secreted EV cargo proteins (Fig. 1A). Epithelial explants were treated daily with either (i) PBS (control), (ii) fallopian tube sEVs (FT240 derived, 1.5 × 10^9^ particles/day), or (iii) HGSOC sEVs (OVCAR3 derived, 1.5 × 10^9^ particles/day). FT-derived EVs were included as a concentration-matched control to account for cargo-independent effects of increased EV abundance as elevated EV levels—regardless of cargo—can modulate cellular pathways and repress exocytosis (31). Because of tissue size limitations, we were unable to test the effects of HGSOC-derived EVs from multiple HGSOC cell lines. However, previous work in our lab (26) has shown high similarity between protein content in EVs derived from HGSOC cell lines (Supplementary Fig. S1A and S1B), suggesting that EVs from a single cell line model can provide a useful starting point for the study of HGSOC. To ensure physiologically relevant dosing, we used our previous work (18) to estimate that hFTE explants produce ∼5.3 × 10^8^ EVs/day (range, 5.67 × 10^7^ to 1.06 × 10^9^) over a 6-day culture period (Supplementary Fig. S1). Adjusting for EV loss during purification, a dosage of 1.5 × 10^9^ particles/day was chosen as a realistic starting point for EV treatment.

To study both short- and long-term consequences of EV insult, we exposed the tissue to sEVs for either 1 day (*n* = 13 samples) or repeatedly over 14 days (*n* = 10 samples; Fig. 1A). We chose to study two time points; although 14 days of culture are needed to collect sufficient EVs for proteomics analysis, the 1-day culture is optimal for studying the immediate signaling effects of EV stimulation. At the end of the treatment period, fallopian tissue samples were collected for both 1- and 14-day conditions and analyzed on the GeoMx DSP using the CTA panel. The CTA panel permits analysis of the expression of ∼1,800 cancer-related genes (about 10% of the human transcriptome) using a set of specially constructed probes that bind to mRNA targets present in the tissue. Information on the quality control analysis of the dataset is presented in Supplementary Fig. S2.

Conditioned medium samples were also collected from the 14-day culture conditions and sEVs were purified using differential ultracentrifugation. Samples with sufficient protein (at least 8 μg, *n* = 15) were analyzed using label-free mass spectrophotometry to identify changes in the EV proteome.

IHC staining for PAX8 and FOXJ1, markers for secretory cells and ciliated cells, confirmed the presence of the two major epithelial cell populations after FTE cultured on PREDICT-MOS for 14 days (Fig. 1B). Because secretory cells are believed to be the cell of origin of HGSOC and secretory cell expansion is a risk factor for serous neoplasia (5), we chose to use the GeoMx platform to segment the hFTE into ciliated and secretory cell types and analyze transcriptomic changes in these cell populations separately. An example of IF staining and automatic segmentation of both cell types is shown in Fig. 1C.

### Purification of sEVs, characterization, and confirmation of sEV uptake in tissue

**Figure 2:**
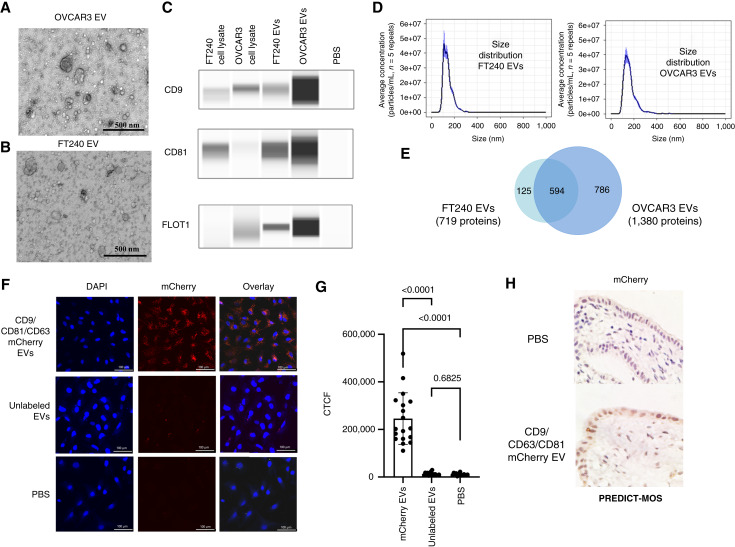
Purified EV characterization and confirmation of sEV uptake in tissue. **A** and **B,** transmission electron micrograph of sEVs from OVCAR3 (**A**) and FT240 (**B**) cells under 30,000× magnification. **C,** Western blots comparing the expression of sEV markers CD9, CD81, and FLOT1 in FT240 and OVCAR3 cell lysates and purified sEVs. Purified sEVs show enrichment of EV markers. **D,** Size distribution of OVCAR3 and FT240 sEVs determined using NTA. **E,** Venn diagram showing the number of proteins identified in FT240 and OVCAR3 sEVs as well as the overlap between samples. **F,** Confocal fluorescent imaging of mCherry-labeled FT240 cell–derived sEV uptake (2 μg) in fallopian tube cells. **G,** Bar plot quantifying uptake of mCherry in fallopian tube cell line (**F**). Each point represents an individual cell. **H,** IHC staining for mCherry after human fallopian tube tissue incubation with 1.51E10 mCherry-labeled FT240 cell–derived sEV for 24 hours.

We selected cell line models of HGSOC (OVCAR3) and, as a control, immortalized, non-tumorigenic hFTE (FT240) as the source of EVs for these experiments. Conditioned medium was collected from these cell lines and differential ultracentrifugation was used to purify sEVs. To validate successful EV purification, we used transmission electron microscopy to demonstrate that both samples show characteristic EV morphology (Fig. 2A and B). To confirm that the observed vesicles were EVs, purified EV samples and corresponding cell lysates were prepared with equal amounts of total protein and then analyzed via Western blotting, probing for established EV markers CD9, CD81, and FLOT1 (Fig. 2C; uncropped images are shown in Supplementary Fig. S3). The results showed that EV samples were enriched for characteristic EV markers compared with the parental cell line. NTA was used to estimate particle concentration and size distribution (Fig. 2D). All purified samples had typical EV size ranges of 100 to 200 nm.

To characterize the proteins present in the sEV samples used for treatment, we used label-free mass spectrophotometry following a data-dependent acquisition method to analyze detectable proteins present in both OVCAR3 and FT240 EV samples. A total of 719 proteins were identified in the FT240 EVs and 1,380 proteins in the OVCAR3 EVs (Fig. 2E). Both samples show a large overlap of common proteins (594 total). For a full list of proteins detected, please see Supplementary Data File 1—OVCAR3-FT240 EVs Proteomic Profile.

The EVs were diluted to the appropriate concentration in filtered PBS, and 50 μL was added daily to the medium intake well of the microfluidic device. In the 1-day cultures, EVs were added at the beginning of the 24-hour period, whereas in the 2-week long-term cultures, EVs were added once daily. To track the internalization of sEVs in the fallopian tube tissue, we generated an FT240 cell line with mCherry conjugated to EV markers CD9, CD63, and CD81, resulting in very bright endogenously labeled EV particles for tissue uptake experiments. We first validated uptake in fallopian tube cell lines cultured in eight-well plates (Fig. 2F). Cell lines were treated with 2 μg of mCherry FT240 EVs for 1 day and then stained with DAPI. Confocal fluorescence microscopy showed clear mCherry signal in cells treated with mCherry-EVs versus PBS control over a 1-day period.

To further validate uptake in tissue, human fallopian tube tissue explants were incubated with 1.5 × 10^10^ mCherry-EVs for 24 hours. Because of issues with tissue autofluorescence masking mCherry signal, we used IHC staining as an alternative detection method. IHC probing for mCherry showed a signal in the EV-treated fallopian tube tissue, with no staining in the PBS-treated control, confirming the uptake of sEVs (Fig. 2G).

### Transcriptomic profiles of OVCAR3 EV–treated hFTE reveal upregulation of immune-related transcripts and pathways

We used the GeoMx DSP to identify transcriptomic changes induced by EV insult in hFTE tissue explants. To focus on transcripts relevant to ovarian cancer, we used the CTA panel. The CTA panel contains ∼1,800 genes (approximately 10% of the full human transcriptome) selected for their relevance to human cancer development and progression.

After overnight hybridization with CTA probes, hFTE slides were stained for markers of secretory (PAX8) and ciliated (FOXJ1) cells to distinguish epithelial cell types. ROIs were drawn to capture the epithelium, and each ROI was auto-segmented into secretory and ciliated segments based on staining (Fig. 1C). To avoid differences in slide staining and probe conjugation, all tissue samples from the same individual (including PBS-, OVCAR3 EV–, and FT240 EV–treated tissue) were placed on the same slide and analyzed together. The final output was transcript count for each segment (ciliated or secretory) under three treatment conditions (PBS, FT240 EV, and OVCAR3 EV).

In the 1-day EV treatment cohort, we collected 319 segments from fallopian tube tissue explant samples from 13 unique individuals (19–56 years of age). All tissue samples were removed for non-cancer indications and were pathologically normal.

In the 14-day EV treatment cohort, we collected 295 segments from nine patients (22–51 years of age). Patient cohort characteristics for both the 1-day and 14-day treatment samples are shown in Supplementary Table S1, whereas Supplementary Table S2 records the number of segments collected for each patient, treatment condition, and cell type.

After the initial quality control as described under methods, 224 segments (70%) remained in the 1-day cohort and 183 segments (62%) in the 14-day treatment cohort. Following Q3 normalization, we identified 1,139 transcripts with detectable expression in the fallopian tube. The quality control analysis is shown in Supplementary Fig. S2.

After normalization, we performed differential gene expression analysis comparing the secretory and ciliated segments for the day-1 (Supplementary Fig. S4A) and day-14 (Supplementary Fig. S4B) cohorts to verify that segmentation was successful. As expected, markers of secretory cells (PAX8) were significantly upregulated (Supplementary Fig. S4C and S4D) in secretory segments (1-day: *P* = 0.0026; 14-day: *P* = 1.6 × 10^−5^) whereas markers of ciliated cells (FOXJ1) were significantly upregulated (Supplementary Fig. S4C and S4D) in ciliated segments (1-day: *P* < 2.2 × 10^−16^; 14-day: *P* = 2 × 10^−14^). The full list of DEGs identified in the two segments is provided in Supplementary Data File S2. After validating that ciliated and secretory segmentation was successful, we identified transcripts that were differentially expressed (*P* < 0.05, |log_2_ FC| > 0.5) between the two treatment conditions.

**Figure 3:**
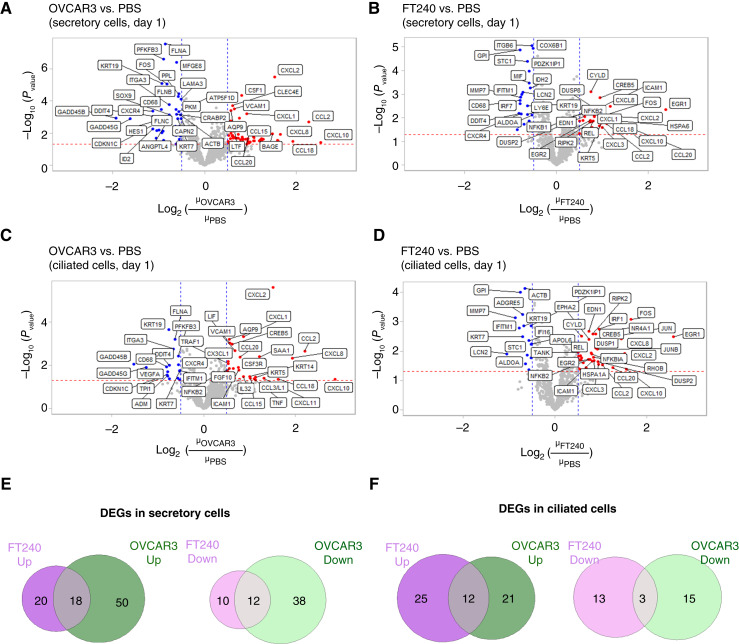
Transcriptomics analysis of hFTE after short-term EV exposure (1 day). **A** and **B,** Volcano plots showing differentially expressed transcripts in secretory cells in (**A**) OVCAR3 EV–treated and (**B**) FT240 EV–treated hFTE compared with PBS controls. **C** and **D,** Volcano plots showing differentially expressed transcripts in ciliated cells in (**C**) OVCAR3 EV–treated and (**D**) FT240 EV–treated hFTE compared with PBS controls. **E** and **F,** Venn diagrams comparing DEGs induced in (**E**) secretory cells and (**F**) ciliated cells by OVCAR3 EV treatment compared with FT240 EV treatment.

In the short-term treatment dataset (Fig. 3), for secretory cells, we identified 68 genes upregulated by OVCAR3 sEVs (Fig. 3A) and 38 genes upregulated by FT240 EVs (Fig. 3B). Of these, 50 were uniquely upregulated only by OVCAR3 sEVs (Fig. 3E) and not by the FT240 EV control, indicating that they were specifically induced by cancer EVs. Treatment with OVCAR3 sEVs also downregulated 50 transcripts in secretory cells, 38 of which were uniquely downregulated by OVCAR3 sEVs (Fig. 3E). In ciliated cells (Fig. 3C and D), 21 genes were uniquely upregulated by OVCAR3 EVs and 15 genes were uniquely downregulated (Fig. 3F). In general, secretory and ciliated cells showed similar upregulated and downregulated genes although the FC detected in ciliated cells tended to be smaller.

**Figure 4:**
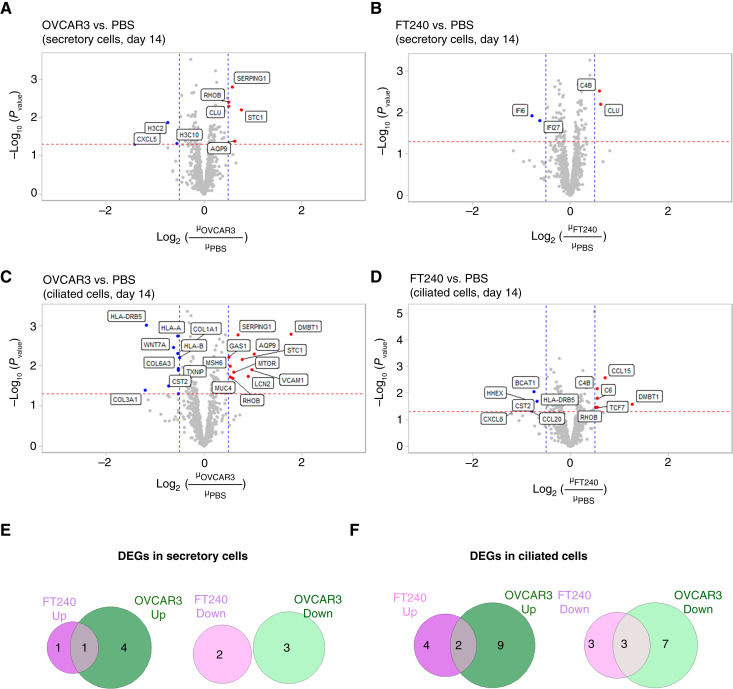
Transcriptomics analysis of hFTE after long-term EV exposure (14 days). **A** and **B,** Volcano plots showing differentially expressed transcripts in secretory cells in (**A**) OVCAR3 EV–treated and (**B**) FT240 EV–treated hFTE compared with PBS controls. **C** and **D,** Volcano plots showing differentially expressed transcripts in ciliated cells in (**C**) OVCAR3 EV–treated and (**D**) FT240 EV–treated hFTE compared with PBS controls. **E** and **F,** Venn diagrams comparing DEGs induced in (**E**) secretory cells and (**F**) ciliated cells by OVCAR3 EV treatment compared with FT240 EV treatment.

In the long-term treatment samples (Fig. 4), we observed fewer DEGs. In secretory cells, OVCAR3 treatment uniquely upregulated four genes and downregulated three (Fig. 4A, B and E). In ciliated cells, OVCAR3 EVs uniquely upregulated nine genes and downregulated seven (Fig. 4C, D and F). Notably, in both cell types, three genes (*AQP9*, *STC1*, and *SERPING1*) were upregulated. Two genes (*VCAM1* and *AQP9*) were consistently upregulated under both long-term and short-term conditions (and none were consistently downregulated). It is possible that negative feedback to repeated EV stimulation resulted in fewer DEGs in the long-term dataset. A full list of differentially expressed transcripts between treatment conditions is provided in Supplementary Data File S3. We attempted to validate EV-induced upregulation of the chemokine CCL2 at the protein level in fallopian tube cell lines (Supplementary Fig. S5), but did not observe a change in CCL2 secretion, suggesting that immortalized cell lines devoid of stromal components do not fully recapitulate the FTE and do not necessarily have the same inflammatory response to EV signaling. A similar result was obtained using qPCR to detect changes in the transcripts in EV-treated cell lines (Supplementary Fig. S6). Some cell lines responded to EV treatment by upregulating inflammatory transcripts, but others did not, emphasizing that *ex vivo* tissue explants may have substantially different responses to EVs that are not captured by cell line models. It is also possible that growth of cells in a monolayer on plastic, absent in their normal underlying stroma layer as in our tissue culture, results in activation of chemokine or IFN pathways (as seen in Supplementary Fig. S5, there is high expression of CCL2 even in untreated control). Future fallopian tube organoid models incorporating stromal components may therefore be needed to create a model system that fully recapitulates the tissue microenvironment.

To investigate whether insult with OVCAR3 EVs also induced DNA damage in hFTE, we stained hFTE samples that had been exposed to EVs for short- and long-term periods for 53BP1, a marker of DNA damage (Supplementary Figs. S7 and S8). Preliminary results showed no notable differences between samples, suggesting that OVCAR3 EVs are unlikely to significantly affect certain markers of DNA damage. We also investigated whether OVCAR3-derived EVs could cause downregulation of PAX2—a common change seen in secretory cell outgrowths. IF staining for PAX2 showed reduction in expression in OVCAR3-treated samples relative to controls (Supplementary Fig. S9).

### Ovarian cancer EVs activate immune signaling pathways

**Figure 5:**
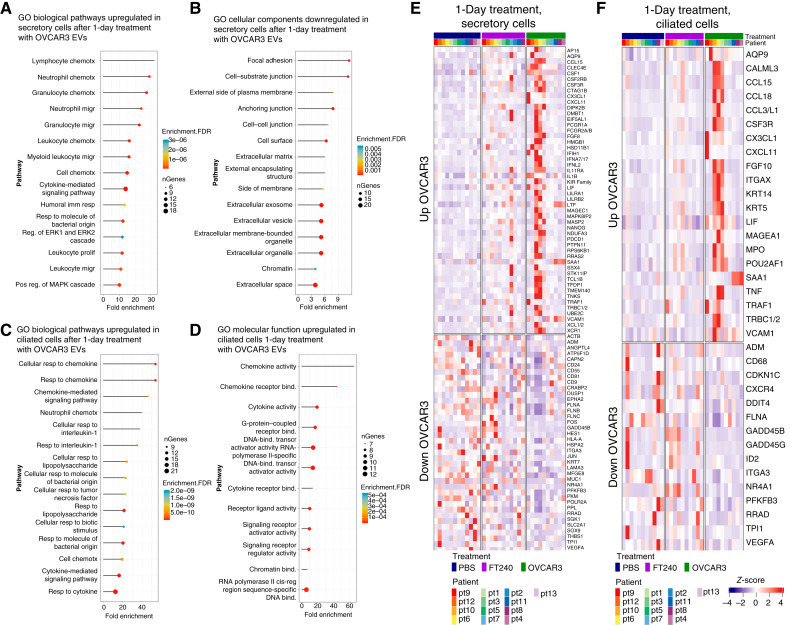
EV treatment with ovarian cancer EVs induced immune responsive and chemokine signaling pathways. **A** and **B,** GO analysis showing pathways upregulated (**B**) and downregulated (**C**) by OVCAR3 EVs in secretory cells. **C** and **D,** GO analysis showing (**C**) biological pathways and (**D**) molecular functions upregulated in ciliated cells by OVCAR3 EVs. **E** and **F,** Heatmap showing transcripts upregulated and downregulated by OVCAR3 EVs in (**E**) secretory cells and (**F**) ciliated cells following 1 day of treatment.

To identify potential cellular reprogramming induced by cancer-derived sEVs, we further analyzed the transcripts that were uniquely upregulated or downregulated by OVCAR3 EVs compared with controls in the short-term treatment dataset. We used GO biological pathway analysis to identify enriched pathways. In secretory cells, OVCAR3 EV treatment strongly upregulated immune-related pathways (Fig. 5A), including pathways related to leukocyte chemotaxis, granulocyte chemotaxis, cytokine-mediated signaling pathways, and leukocyte proliferation. GO cellular component analysis (Fig. 5B) showed that treatment with sEVs also resulted in secretory cell downregulation of transcripts related to focal adhesion and EVs, suggesting that stimulation with EVs results in a downregulation of EV production by secretory cells. Similar chemokine and immune-related biological pathways are upregulated in ciliated cells. GO Molecular Function analysis showed that pathways related to response to chemokine, neutrophil chemotaxis, and G-protein–coupled receptor binding were also significantly upregulated in ciliated cells (Fig. 5C and D). The equivalent GO analysis for FT240 EVs may be found in Supplementary Figs. S10 and S11; we also performed Reactome and Kyoto Encyclopedia of Genes and Genomes pathway analysis, which likewise showed immune-related pathways upregulated by OVCAR3 EVs (Supplementary Fig. S12 and S13).

A heatmap comparison of the top genes upregulated and downregulated by EVs in secretory (Fig. 5E) and ciliated cells (Fig. 5F) showed significant shifts in immune-related genes induced by OVCAR3 EVs in comparison with the PBS control (see Supplementary Fig. S14 for unsupervised clustering of the same heatmaps). Interestingly, FT240 EVs, despite being putatively “normal EVs,” also induce a similar, although substantially weaker, response in many of the same genes as the OVCAR3 EVs. As the FT240 cell line immortalization process induced genetic alterations comparable with early preneoplastic lesions (short hairpin RNA knockdown of p53, expression of mutant *CDK4*^R24C^ to inhibit pRb, and exogenous expression of hTERT), this may indicate that the EVs produced by cells without p53 and Rb induce alterations in the immune landscape very early in the development of neoplastic cells in the fallopian tube.

### HGSOC EVs upregulate genes activated in early precursors

Recent work has used the GeoMx spatial transcriptomic platform to analyze HGSOC precursor lesions at different stages of development, comparing p53 signature and STIC lesions to adjacent normal epithelia and invasive cancer (27). The authors demonstrated that type II IFN pathway genes were upregulated during progression; we observed that HGSOC EVs upregulate transcripts in this pathway (*NFKB1*, *NFKB2*, *CXCL10*, and *CXCL11*; Supplementary Fig. S15). We searched for other potential overlaps and found that *CXCL2*, *LIF*, *UBE2C*, *KRT5*, *ICAM1*, and *CXCL8* were significantly upregulated in STIC lesions identified in patients with cancer compared with adjacent normal epithelium (Supplementary Fig. S16).

### Ovarian cancer EVs cause a shift in cargo of secondary-release hFTE-derived EVs

**Figure 6:**
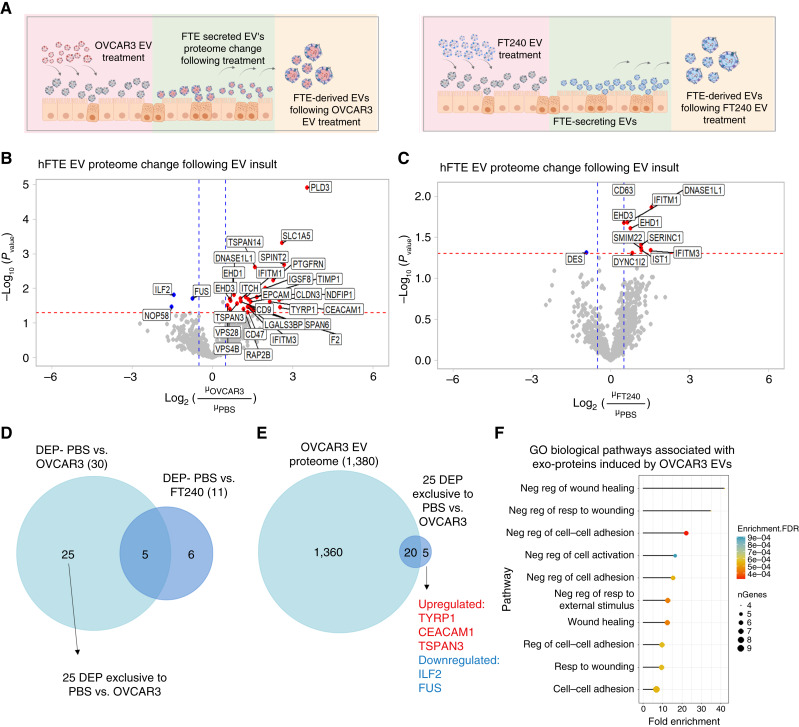
Fallopian tube alters sEVs protein cargo after treatment with OVCAR3 EVs for 14 days. **A,** Diagram showing EV treatment of hFTE tissue and production of secondary-release EVs. **B,** Volcano plot showing proteomic changes in EVs derived from tissues treated with OVCAR3 EVs compared with PBS control. **C,** Volcano plot showing proteomics changes in EVs derived from tissues treated with FT240 EVs compared with PBS-treated controls. **D,** Venn diagram showing identification of 25 proteins exclusively upregulated by OVCAR3 EVs only. **E,** Venn diagram showing detection of DEPs not present in original OVCAR3 EVs in hFTE-derived EVs. **F,** GO biological pathway analysis of DEPs in hFTE-derived secondary-release EVs following stimulation with OVCAR3 EVs.

For the long-term EV exposure cultures, we collected conditioned medium daily over the 2 weeks when tissue was exposed to EVs (Fig. 6A). The PREDICT intakes 1.5 mL of fresh medium (with or without sEV supplemented) per day and releases 1.5 mL of conditioned medium into a sterile collection well over the same period. Approximately 21 mL of medium was collected over the 2-week culture period. Following collection, the samples were purified using ultracentrifugation, and those with sufficient EV protein yield were sent for analysis. Five samples had sufficient yield to perform mass spectrophotometry under all three conditions, attributable to differences in patient heterogeneity, tissue conditions, and yield between different centrifugation runs. The patient cohort characteristics of these samples are shown in Supplementary Table S1.

Following proteomic analysis, we compared the expression of proteins between the control and EV-treated hFTE-derived EV samples. We found 30 differentially expressed proteins (DEP) in the OVCAR3 treatment samples (Fig. 6B) and 11 DEPs in the FT240-sEV–treated samples (Fig. 6C). To identify proteins uniquely upregulated only by OVCAR3-sEVs, we compared the two datasets (Fig. 6D) and identified 25 proteins that were upregulated only by OVCAR3-sEVs. As the tissue may not completely take up treated OVCAR3 sEVs, some of the excess OVCAR3 sEVs from the treatment may co-isolate with the hFTE-derived sEVs. We compared the 25 uniquely upregulated proteins to the full OVCAR3 EV proteome identified previously and found that five of these DEPs (TYRP1, CEACAM1, TSPAN3, ILF2, and FUS) were unique to hFTE-derived EVs and were not detected in OVCAR3-derived sample (Fig. 6E). Finally, we used GO biological pathway analysis to identify enriched pathways associated with the 25 DEPs modified by OVCAR3-sEV treatment (Fig. 6F). The most enriched pathways included those related to the negative regulation of wound healing, negative regulation of cell–cell adhesion, and negative regulation of cell activation.

### IHC analysis validates that EV-induced transcriptomic changes result in corresponding shifts in protein expression in the hFTE

**Figure 7:**
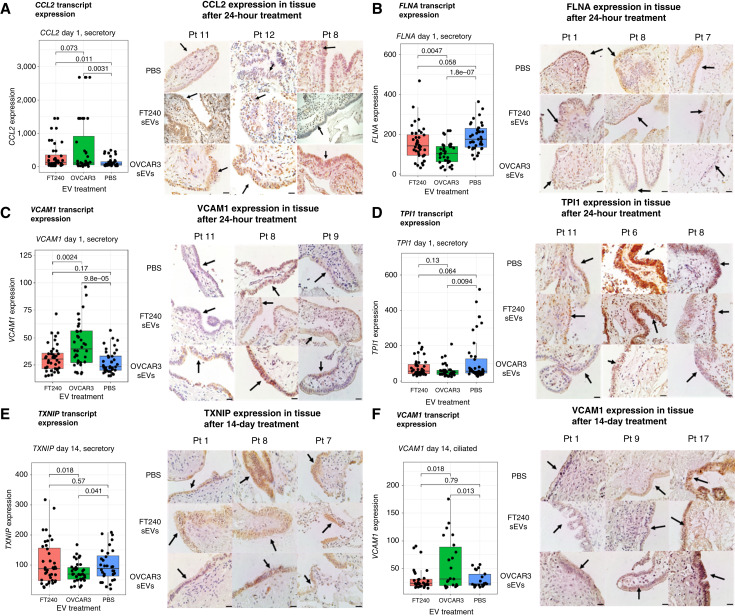
Validation of 1-day and 14-day treatment transcriptomics data using IHC. **A,** Left, *CCL2* transcript expression in OVCAR3-, FT240-, and PBS-treated secretory cells after 1 day. Right, IHC staining for upregulated CCL2 in FTE from three individual patients. **B,** Left, *FLNA* transcript expression in OVCAR3-, FT240-, and PBS-treated secretory cells after 1 day. Right, IHC staining for downregulated FLNA in FTE from three individual patients. **C,** Left, *VCAM1* transcript expression in OVCAR3-, FT240-, and PBS-treated secretory cells after 1 day. Right, IHC staining for upregulated VCAM1 in FTE from three individual patients. **D,** Left, *TPI1* transcript expression in OVCAR3-, FT240-, PBS-treated secretory cells after 1 day. Right, IHC staining for downregulated protein TPI1 in FTE from three individual patients. **E,** Left, *TXNIP* transcript expression in OVCAR3-, FT240-, and PBS-treated secretory cells after 14 days. Right, IHC staining for downregulated TXNIP in FTE from three individual patients. **F,** Left, *VCAM1* transcript expression in OVCAR3-, FT240-, and PBS-treated ciliated cells after 14 days. Right, IHC staining for upregulated VCAM1 in FTE from three individual patients. In all boxplots, each point represents normalized transcript abundance measured in a single segment. Scale bar, 20 μm; arrows indicate epithelium.

To investigate whether the expression of the upregulated or downregulated transcripts also resulted in changes in protein expression, we used IHC to evaluate the expression of CCL2, VCAM1, FLNA, TPI1, and TXNIP in the tissues after either 1-day or 14-day exposure. These transcripts were selected because they were either uniquely upregulated by OVCAR3 sEVs treatment but not by FT240 sEVs treatment or because they had the highest FC in expression after OVCAR3 sEVs exposure. Three fallopian tube samples treated with PBS or OVCAR3 sEVs were stained for the above protein targets. Box plots indicate the corresponding transcript expression from the spatial transcriptomics data. In tissue after 24 hours of OVCAR3 sEVs exposure, there was a stronger brown staining of FTE cells for CCL2 and VCAM1 compared with the PBS-treated group, indicating the that the level of CCL2 and VCAM1 has a trend of increased expression (Fig. 7A and C). In contrast, tissue treated with OVCAR3 sEVs for 24 hours showed less intense staining for FLNA and TPI1, confirming that FLNA and TPI1 have decreased expression after treatment (Fig. 7B and D). VCAM1 and TXNIP protein levels varied among different patients. After 14-day OVCAR3 sEVs exposure, two of three patient fallopian tube samples showed a decreasing trend of TXNIP expression and a trend of increasing expression of VCAM1 in tissue (Fig. 7E and F). These findings demonstrate that the transcriptomic changes induced by OVCAR3 sEVs are reflected at the protein level, providing further evidence of their functional impact on hFTE cells.

## Discussion

### Short term stimulation of the inflammatory response induced by OVCAR3 EVs

In this study, we showed that EVs from ovarian cancer cells upregulated genes related to the inflammatory response, including immune cell–attracting cytokines (*CCL2*, *CCL3/L1*, *CCL15*, *CCL18*, and *CCL20*), interleukins (*IL1B* and *IL32*), and cell–cell adhesion transcripts (*VCAM1* and *ICAM1*).

Upregulation of these transcripts may be related to *NFKB* pathway activation as NF-κB subunits (*NFKB1* and *NFKB2*) were upregulated.

### Chemokine expression induced by OVCAR3 EVs

The upregulated chemokines can attract a diverse range of innate and adaptive immune cells, for example, CCL2 attracts tumor-associated macrophages, CXCL10/11 attracts CD8^+^ T cells, CCL20 attracts dendritic cells, and CXCL5 attracts neutrophils (32). These chemokines have both pro- and antitumor effects reported in ovarian cancer.

On the pro-tumor side, CCL2 (MCP-1), a potent monocyte chemoattractant (33), is correlated with poor overall survival (34) and contributes to ovarian cancer progression through recruitment of M2 macrophages (35), promotion of tumor metastasis (36), and promotion of chemotherapy resistance (34). Other chemokines potentially involved in cancer progression include CCL18, which promoted ovarian cancer migration (37), CCL20, which promoted both metastasis (38) and chemotherapy resistance (39), and CXCL8 (IL-8), which may contribute to peritoneal metastasis (40). Activation of some of these chemokines in the surrounding microenvironment could plausibly lead to reprogramming of nearby niches within the ovary or peritoneal cavity, perhaps creating a favorable microenvironment for metastases.

However, not all upregulated chemokines are likely to promote ovarian cancer progression. High expression of CXCL2, CXCL10, and CXCL11 is correlated with favorable prognoses in ovarian cancer (41–43). Both CXCL10 and CXCL11 mediate CD8^+^ T-cell responses (32), and CXCL10 promotes C8^+^ T-cell activation in ovarian cancer (44).

It would be oversimplifying to qualify this diverse combination of immune-related transcripts as pro– or anti–ovarian cancer. The actual consequences of chemokine signaling are context dependent and influenced by ongoing interactions between the stroma, immune system, and cancer. Our microfluidic culture medium did not support survival of resident immune cells for long periods of time and would require optimization to incorporate immune cell recruitment and infiltration. Although it is clear that short-term stimulation with HGSOC-EVs can trigger a strong immune response in neighboring tissues, the exact consequences of EV-induced immune signaling will require further investigation using other models such as syngeneic mouse models.

### Metabolic genes involved in aerobic glycolysis were downregulated by OVCAR3 EV treatment

The metabolic genes *TPI1*, *PKM*, *ATP5F1D*, and *PFKFB3* were downregulated following short-term OVCAR3-EV stimulation. *TPI1*, or triosephosphate isomerase, catalyzes the interconversion of dihydroxyacetone phosphate and glyceraldehyde 3-phosphate (45). Downregulation of this gene prevents dihydroxyacetone phosphate from being converted to glyceraldehyde 3-phosphate and entering the glycolysis pathway. PKM encodes PKM1 and PKM2, which are involved in the conversion of phosphoenolpyruvate to pyruvate (46), whereas *ATP5F1D* encodes a subunit of mitochondrial ATP synthase (47). Finally, *PFKFB3* encodes 6-phosphofructo-2-kinase (PFK-2), an enzyme involved in regulating the rate of glycolysis and gluconeogenesis (48). PFK-2 controls the concentration of fructose-2,6-bisphosphate, which regulates the glycolysis rate by allosteric activation of 6-phosphofructo-1-kinase (48). PFK-2 is often overexpressed in hypoxic environments and tumors, leading to increased glycolysis.

Together, downregulation of these genes suggests a possible metabolic shift away from glycolysis and toward alternative pathways (e.g., oxidative phosphorylation). Notably, this is the opposite of the classical Warburg effect, in which cancer cells preferentially use the glycolysis pathway even in the presence of oxygen. Inhibition of aerobic glycolysis in tumor-neighboring cells could reduce their energy demands, creating a favorable environment with more glucose available for cancer cell growth.

### Immunosuppressive effects following long-term EV stimulus

Many of the effects of short-term EV exposure are significantly reduced in long-term EV exposure although some DEGs (e.g., *VCAM1*) are still persistently upregulated in the long-term exposure dataset.

Many of the proteins found in hFTE-EVs following long-term OVCAR3-EV stimulation suggest long-term inhibition of inflammatory response. For example, one upregulated EV protein, ITCH, is involved in negative regulation of immune signals (49). An activator of ITCH, *NDFIP1* (50), is also upregulated. Together, NDFIP1 and ITCH ubiquitinate TAK1, a kinase that promotes NF-κB activation (51). Upregulation of these proteins therefore suggests negative regulation of NF-κB following long-term OVCAR3-EV stimulation.

Other immune-related proteins found in hFTE-derived EVs include IGSF8, which is frequently overexpressed in tumor cells with antigen presentation defects and acts as a suppressor of NK cells (52). Our group has previously reported IGSF8 as a potential biomarker of ovarian cancer–derived EVs for early detection (26) and it is possible that chronic stimulation with ovarian cancer–derived EVs triggers IGSF8 release from neighboring cells as well. IGSF8 is involved in binding to KIR3DL2 on NK cells (52), inhibiting cytotoxic responses, and may represent a further anti-inflammatory response triggered by long-term EV stimulation.

### Study limitations

We used ovarian cancer–derived EVs to maximize the potential effect on FTE, but because of experimental limitations, i.e., obtaining fresh human fallopian tube tissue samples, we were only able to investigate the effects of FT240- and OVCAR3-derived EVs. Further studies could evaluate the effects of repeated exposure of follicular fluids and associated EVs. Importantly, our prior work demonstrated a high convergence of EV cargo from multiple ovarian cancer cell lines (26). This study investigates epithelial tissue, although the underlying stroma also uptakes EVs, which could influence the results. Further studies could investigate the stroma or evaluate the effects of repeated exposure of follicular fluids and associated EVs. We have previously shown that factors in follicular fluid increase cell proliferation and stimulate cell adhesion in spheroid models, (53) and future work on this platform could help elucidate signaling mechanisms specific to follicular fluid EVs. Although we have identified and confirmed that cancer-associated EV insult can alter the proteome of FTE cells, more functional work is needed to understand the potential mechanism. Second, the transcriptomic analyses performed in this study use a targeted panel (CTA) containing ∼1,800 genes rather than whole-transcriptome analysis (∼18,000 genes). The CTA panel was selected for relevance to cancer biology and provides data on the genes most relevant to cancer progression but may exclude genes with previously unidentified pro-tumor functions. Although many of our samples (10 of 18) were collected from patients undergoing salpingectomy for purposes of elective permanent contraception, some salpingectomies were performed as part of procedures for the treatment of other gynecologic disorders (see Supplementary Table S1), which should be kept in mind when interpreting results.

Another limitation of on-chip culture systems is the limited presence of resident immune cells and lack of integration with the full immune system, preventing the study of long-term immune responses. We observe that OVCAR3 EVs initiate a number of inflammatory processes that are downregulated with chronic stimulus, but without exogenous addition of immune cells, it is difficult to understand the dynamic interaction between tumor-derived signals and the immune response.

A final caveat that should be kept in mind is that some EVs spiked into the culture system may be recovered in the conditioned medium. It is difficult to estimate the percentage of treated EVs that are taken up by hFTE explants, and it is possible that some OVCAR3 EVs contribute to proteins measured in proteomics. To mitigate this problem, we searched specifically for markers without detectable expression in OVCAR3 EVs and showed that 20% (five of 25) DEPs detected on hFTE-EVs fall into this category, demonstrating that the microfluidic culture system can identify upregulation of proteins in hFTE-derived EVs that are undetectable in the initial cancer-derived EVs.

In summary, our findings highlight the capacity of EVs to modulate the immune and metabolic landscapes of neighboring cells. Short-term exposure to OVCAR3 EVs triggers robust inflammatory signaling through upregulation of cytokines, chemokines, and immunomodulatory transcripts, potentially promoting both pro- and antitumor effects depending on context. Simultaneously, the downregulation of metabolic pathways could create a supportive metabolic environment for tumor cells and suppress effector immune cell functions. However, long-term stimulation with EVs creates an immunosuppressive effect, with upregulation of inhibitors of NF-κB pathways (ITCH and NDFIP1) and potentially NK-cell immune checkpoint molecules (IGSF8) on hFTE-derived EVs. These findings highlight the significant role of EVs in modifying the immune response of surrounding tissues and altering the cargo of neighboring hFTE-derived EVs in potentially pro-tumor ways.

## Supplementary Material

Supplementary Data File 2 Transcript expression in ciliated vs secretory cells.

Supplementary Data File 3 Comparison of expression in hFTE with different EV treatments.

Supplementary Data File 4 Differential protein expression in hFTE derived eVs.

Supplementary Figure 1 EV proteome comparison and yield analysis.

Supplementary Figure 2 GeoMx DSP quality control analysis.

Supplementary Figure 3 Uncropped western blot images.

Supplementary Figure 4 Validation of segmentation of ciliated and secretory segments in short-term and long-term culture.

Supplementary Figure 5 Measurement of CCL2 production in cell line models of the fallopian tube shows no significant differences following EV exposure.

Supplementary Figure 6 qPCR analysis shows inconsistent responses in FT cell line models to OVCAR3 EV stimulation.

Supplementary Figure 7 EVs from OVCAR3 do not induce detectable DNA damage relative to controls in short term exposure.

Supplementary Figure 8 EVs from OVCAR3 do not induce detectable DNA damage relative to controls in long term exposure.

Supplementary Figure 9 PAX2 expression is downregulated by OVCAR3 EVs

Supplementary Figure 10 GO Biological pathways upregulated by FT240 EVs.

Supplementary Figure 11 GO Biological pathways downregulated by FT240 EVs.

Supplementary Figure 12 KEGG and reactome pathways upregulated and downregulated by OVCAR3 EVs.

Supplementary Figure 13 Kegg and reactome pathways upregulated and downregulated by FT240 EVs.

Supplementary Figure 14 Unsupervised clustering of Figure 5E.

Supplementary Figure 15 Key IFN-γ pathway genes are upregulated by OVCAR3 EV treatment.

Supplementary Figure 16 Transcripts upregulated by OVCAR3 EVs are also upregulated in progression from precursor lesion to invasive cancer.

Supplementary Methods Supplementary Methods

Supplementary Table 1 Patient Cohort Characteristics

Supplementary Table 2 Segments collected using GeoMx DSP before and after quality control

Supplementary Data File 1 OVCAR3-FT240 EV Proteome Profile
