# Supplementary material for: Defining the Ovarian Cancer Precancerous Landscape through Modeling Fallopian Tube Epithelium Reprogramming Driven by Extracellular Vesicles
Source: Cancer Res Commun. 2025 Aug 4;5(8):1266–81. doi: 10.1158/2767-9764.CRC-25-0064 (PMC12319521; doi:10.1158/2767-9764.CRC-25-0064)
Supplement: Supplementary Figure 1 — EV proteome comparison and yield analysis. [file crc-25-0064_supplementary_figure_1_suppsf1.docx]

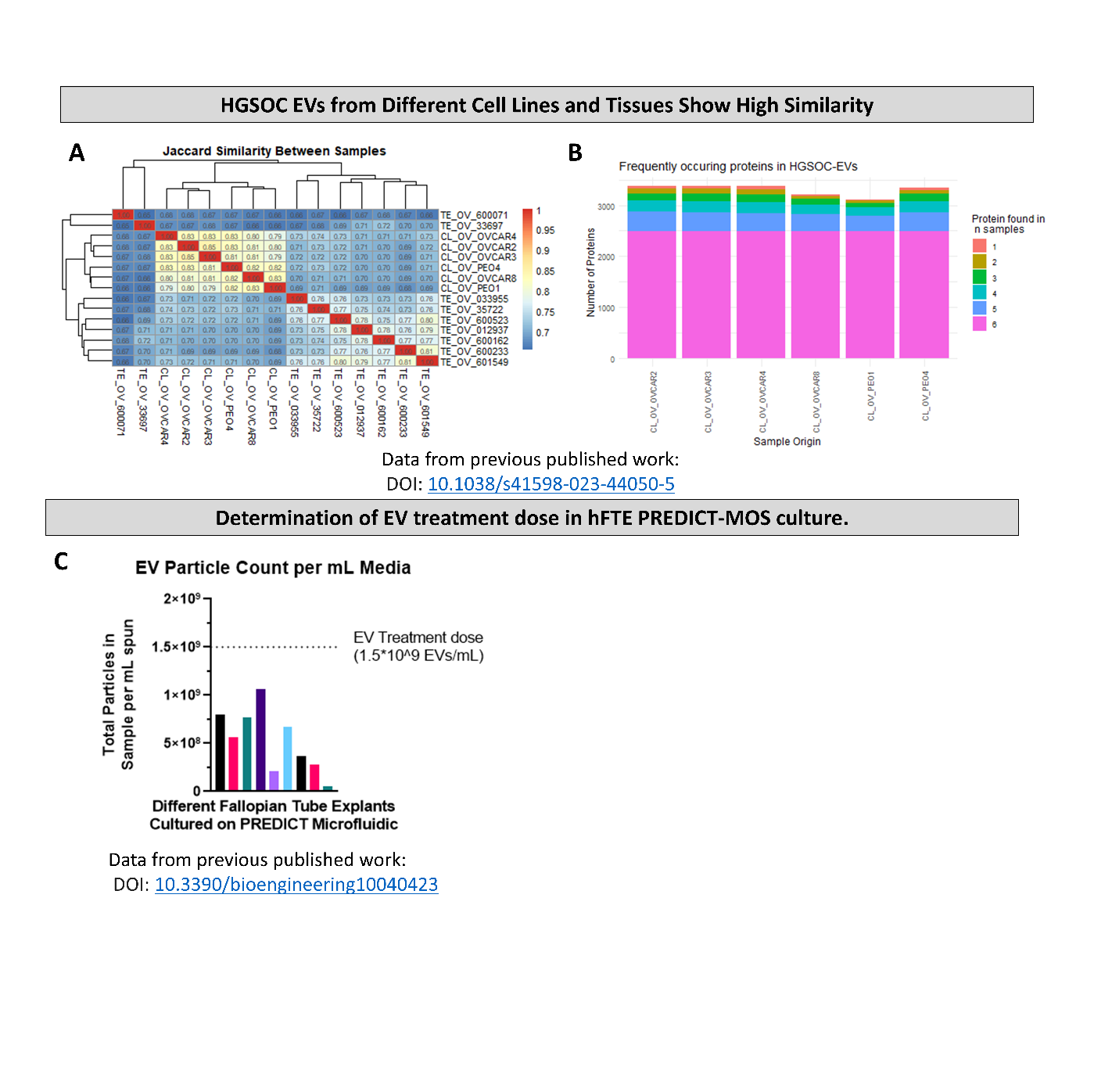


**Supplementary Figure 1.** **EV proteome comparison and yield analysis.**

**A)** Pairwise Jaccard similarity heatmap showing high similarity between High-Grade Serous Ovarian Cancer EV Samples. Heatmap shows Jaccard Score, which is calculated by taking the intersect (# protein in common) and dividing by the union (# protein all) for each sample pair. TE = tissue explant, CL = Cell Line, OV = Ovarian cancer **B)** Stacked bar plot showing 6 HGSOC-EV samples. Color represents the number of samples in which the protein was identified. n= 2491 proteins (pink) were found in all 6 samples. Data in A-B) from previously published work. (DOI: 10.1038/s41598-023-44050-5). **C)** Bar graph comparing EV particle concentrations purified from FT explants cultured on the PREDICT-MOS system and the selected EV treatment dose (dotted line). Data from previously published studies. (DOI: 10.3390/bioengineering10040423).
