# Supplementary material for: Defining the Ovarian Cancer Precancerous Landscape through Modeling Fallopian Tube Epithelium Reprogramming Driven by Extracellular Vesicles
Source: Cancer Res Commun. 2025 Aug 4;5(8):1266–81. doi: 10.1158/2767-9764.CRC-25-0064 (PMC12319521; doi:10.1158/2767-9764.CRC-25-0064)
Supplement: Supplementary Figure 2 — GeoMx DSP quality control analysis. [file crc-25-0064_supplementary_figure_2_suppsf2.docx]

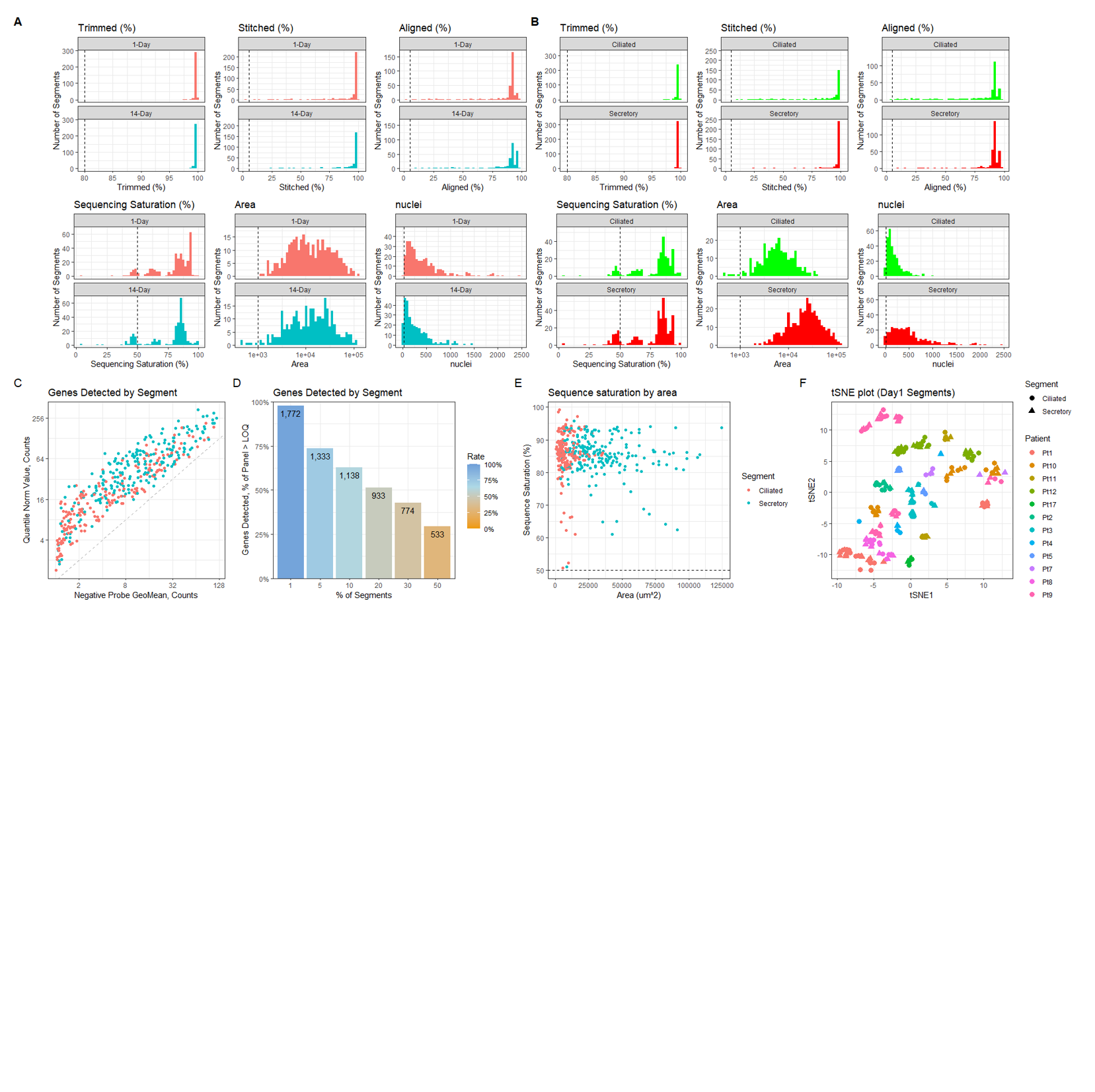


**Supplementary Figure 2. GeoMx DSP quality control analysis.**

**A)** Histograms comparing quality control measurements for segments treated for 1-Day (red) and 14-day (green). The dotted line represents the minimum quality control cutoff (segments below lined removed). **B)** Histograms as in (**A**) but comparing ciliated (green) and secretory (red) segments. The dotted line represents the minimum quality control cutoff (segments below the line are removed). **C)** Scatterplot comparing the negative probe geomean for each segment with the normalized counts. **D)** Barplots showing the percentage of genes above the limit of detection in at least n% of segments (n = 1, 5, 10, etc.) **E)** Scatterplot comparing area to sequence saturation. **F)** TSNE plot showing the clustering of segments post normalization.
