# Supplementary material for: Defining the Ovarian Cancer Precancerous Landscape through Modeling Fallopian Tube Epithelium Reprogramming Driven by Extracellular Vesicles
Source: Cancer Res Commun. 2025 Aug 4;5(8):1266–81. doi: 10.1158/2767-9764.CRC-25-0064 (PMC12319521; doi:10.1158/2767-9764.CRC-25-0064)
Supplement: Supplementary Figure 3 — Uncropped western blot images. [file crc-25-0064_supplementary_figure_3_suppsf3.docx]

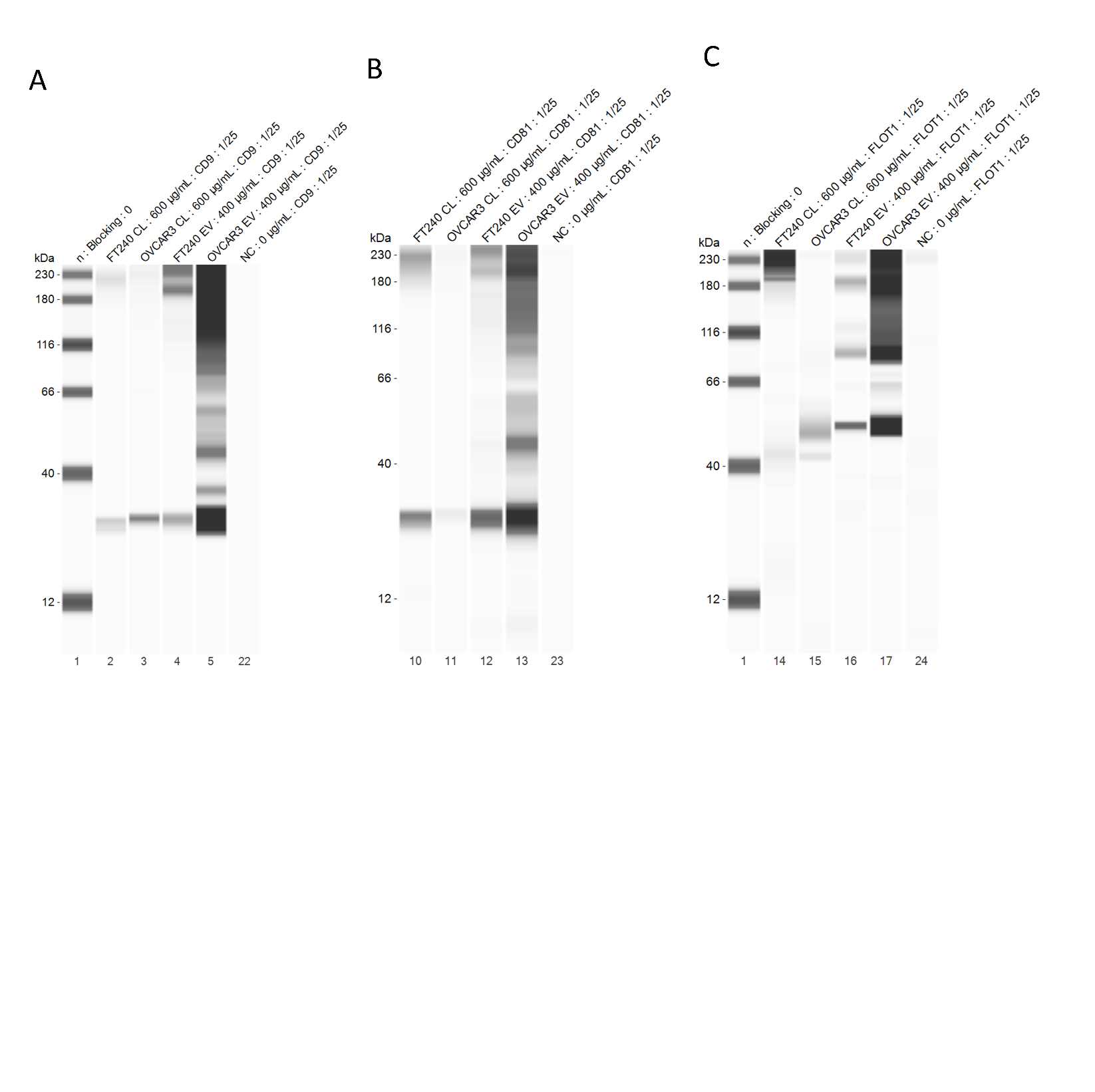


**Supplementary Figure 3. Uncropped western blot images.**

**A-C)** Uncropped western blot images showing staining for common EV markers in either cell lysates (CL) or corresponding purified EV samples (EV), compared to negative control (NC). Loading concentrations and antibody dilutions given in the Figure. **A)** Staining for CD9, **B)** CD81, and **C)** FLOT1.
