# Supplementary material for: Defining the Ovarian Cancer Precancerous Landscape through Modeling Fallopian Tube Epithelium Reprogramming Driven by Extracellular Vesicles
Source: Cancer Res Commun. 2025 Aug 4;5(8):1266–81. doi: 10.1158/2767-9764.CRC-25-0064 (PMC12319521; doi:10.1158/2767-9764.CRC-25-0064)
Supplement: Supplementary Figure 4 — Validation of segmentation of ciliated and secretory segments in short-term and long-term culture. [file crc-25-0064_supplementary_figure_4_suppsf4.docx]

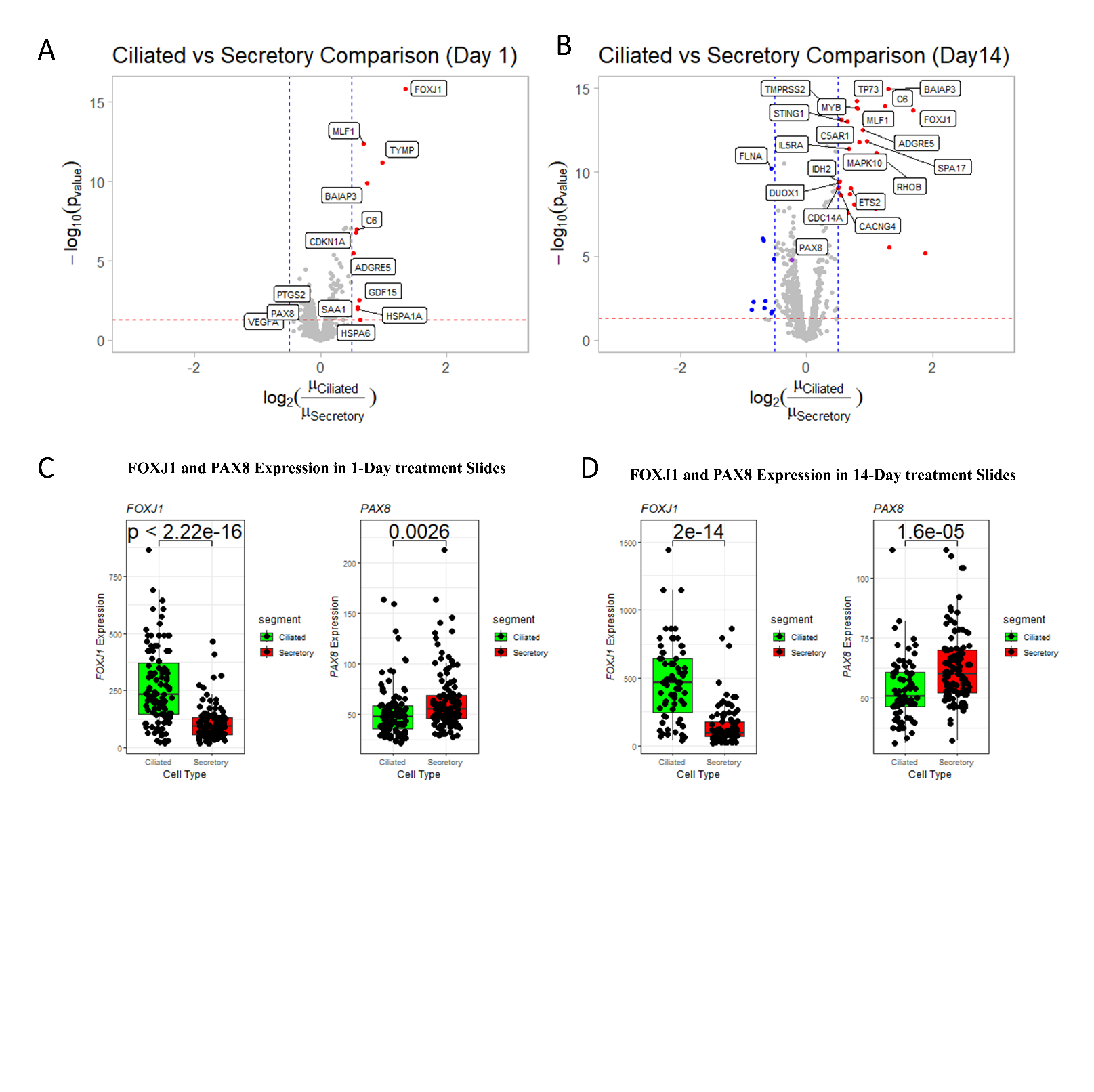


**Supplementary Figure 4. Validation of segmentation of ciliated and secretory segments in short-term and long-term culture.**

**A-B)** Volcano plots showing transcripts overexpressed in ciliated versus secretory segments in **A)** 1-day cultured tissue and **B)** 14-day cultured tissue. **C)** Boxplots showing FOXJ1 and PAX8 expression in ciliated and secretory segments for 1-day cultured tissue. **D)** Boxplots showing FOXJ1 and PAX8 expression in ciliated and secretory segments in 14-day cultured tissues.
