# Supplementary material for: Defining the Ovarian Cancer Precancerous Landscape through Modeling Fallopian Tube Epithelium Reprogramming Driven by Extracellular Vesicles
Source: Cancer Res Commun. 2025 Aug 4;5(8):1266–81. doi: 10.1158/2767-9764.CRC-25-0064 (PMC12319521; doi:10.1158/2767-9764.CRC-25-0064)
Supplement: Supplementary Figure 5 — Measurement of CCL2 production in cell line models of the fallopian tube shows no significant differences following EV exposure. [file crc-25-0064_supplementary_figure_5_suppsf5.docx]

**
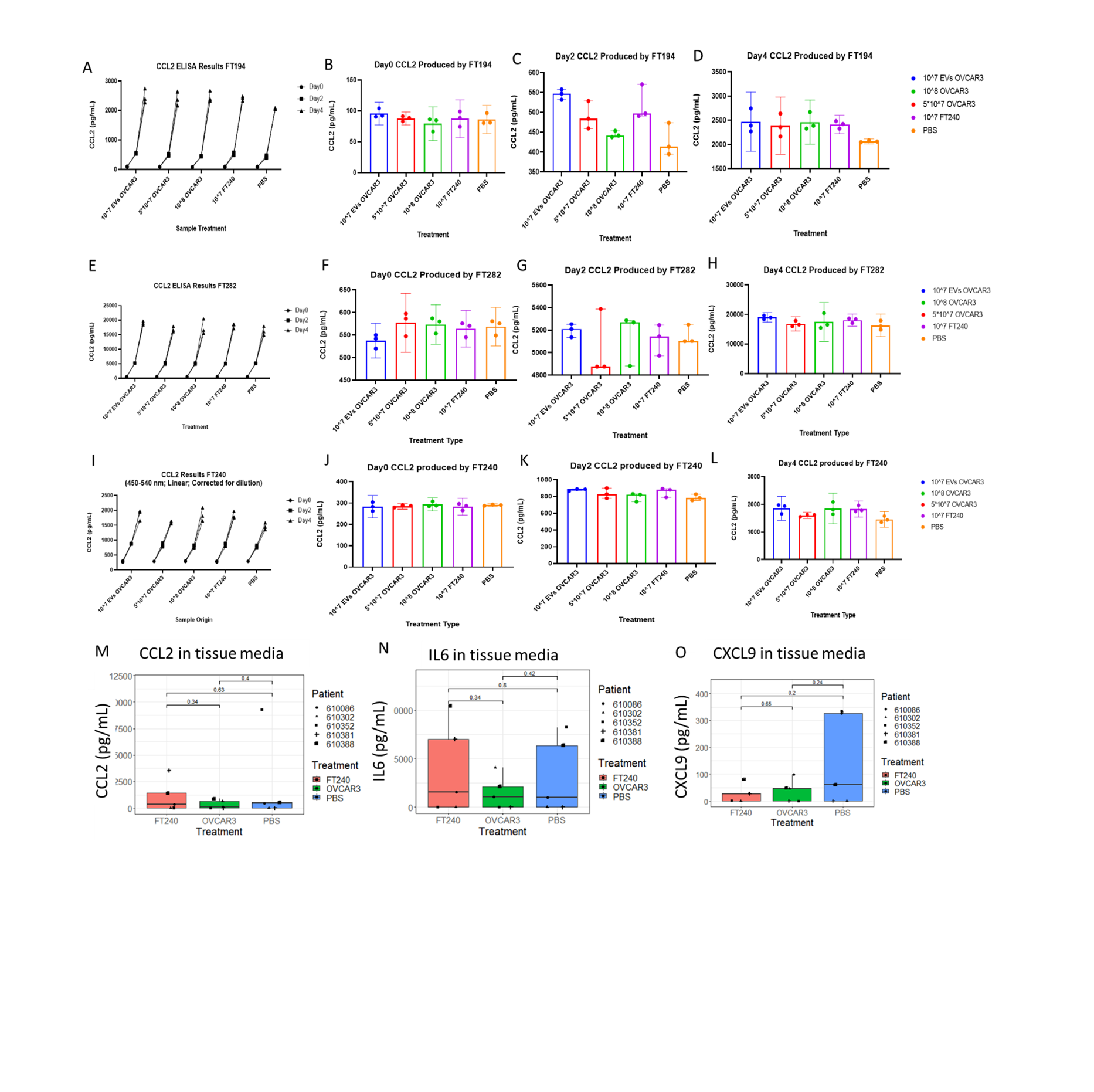
Supplementary Figure 5. Measurement of CCL2 production in cell line models of the fallopian tube shows no significant differences following EV exposure.**

**A)** Time series showing ELISA based measurements of CCL2 in conditioned medium produced by the FT294 cell line stimulated with three different concentrations of OVCAR3 EVs, FT240 EVs, or PBS control. Time points analyzed: Day 0 (before treatment), Day 2, and Day 4. **B-D)** Bar graphs comparing the amount of CCL2 detected in FT194 medium **B)** before EV treatment, **C)** two days after EV stimulation, and **D)** 4 days after EV stimulation. **E-H)** Same as graphs **A-D),** but for the FT282 cell line. **I-L)** Same as graphs **A-D),** but for the FT240 cell line. **M-O)** Boxplots showing selected chemokine expression measured using Luminex assay in tissue conditioned media one day after stimulation with OVCAR3 or FT240 EVs. **M)** CCL2, **N)** IL6, **O)** CXCL9.
