# Supplementary material for: Defining the Ovarian Cancer Precancerous Landscape through Modeling Fallopian Tube Epithelium Reprogramming Driven by Extracellular Vesicles
Source: Cancer Res Commun. 2025 Aug 4;5(8):1266–81. doi: 10.1158/2767-9764.CRC-25-0064 (PMC12319521; doi:10.1158/2767-9764.CRC-25-0064)
Supplement: Supplementary Figure 6 — qPCR analysis shows inconsistent responses in FT cell line models to OVCAR3 EV stimulation. [file crc-25-0064_supplementary_figure_6_suppsf6.docx]

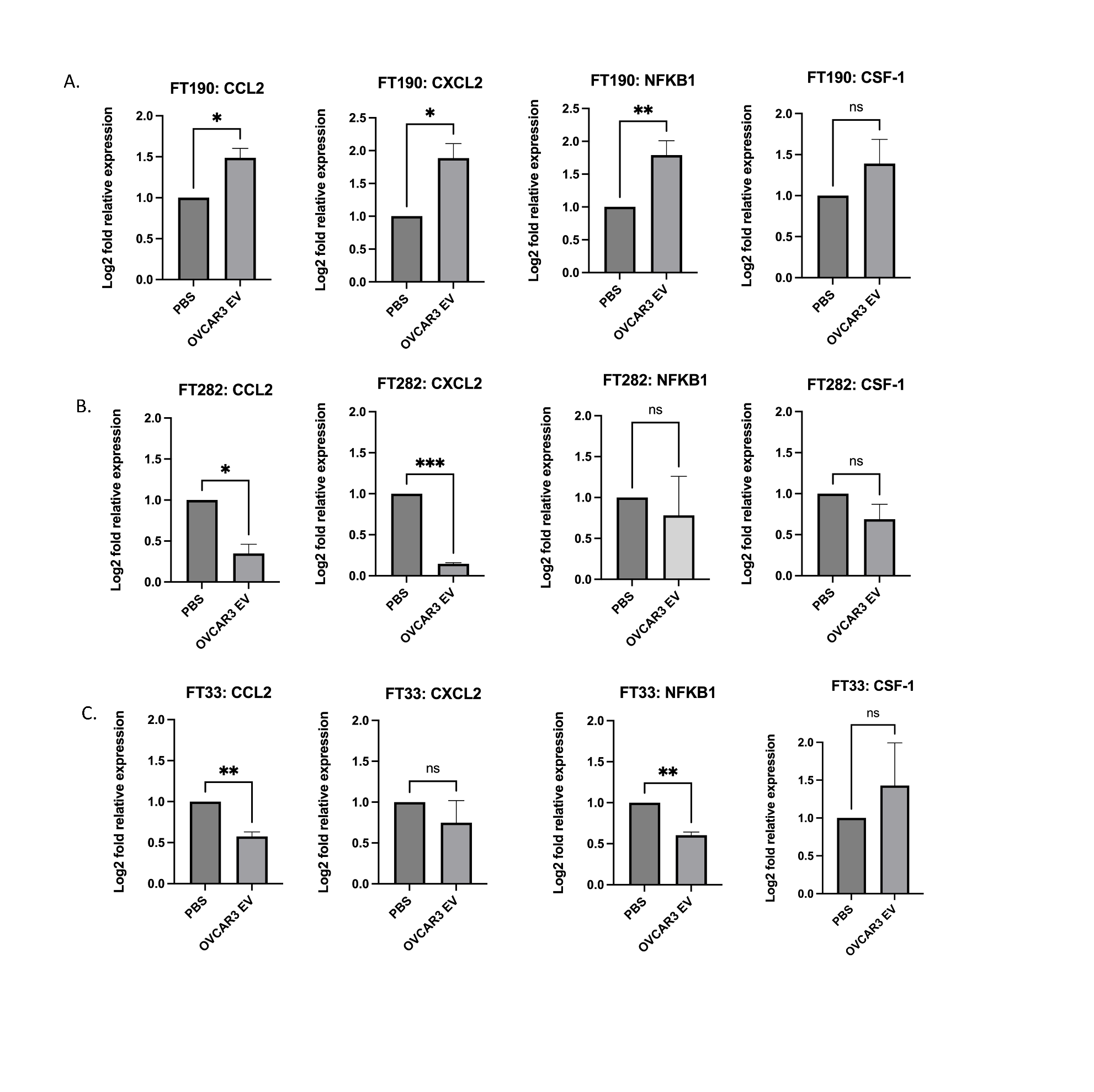
**Supplementary Figure 6. qPCR analysis shows inconsistent responses in FT cell line models to OVCAR3 EV stimulation.**

The expression of upregulated transcripts CCL2, CXCL2, NFKB1 and CSF-1 from spatial transcriptomics analysis are evaluated in three non-tumorigenic fallopian tube cell lines: **A)** FT190, **B)** FT282 and **C)** FT33. Fallopian tube cells were treated with 0.5 µg EV protein/10,000 cells for 24 hours. The qPCR data were analyzed using a non-paired t-test (n=3).
