# Supplementary material for: Defining the Ovarian Cancer Precancerous Landscape through Modeling Fallopian Tube Epithelium Reprogramming Driven by Extracellular Vesicles
Source: Cancer Res Commun. 2025 Aug 4;5(8):1266–81. doi: 10.1158/2767-9764.CRC-25-0064 (PMC12319521; doi:10.1158/2767-9764.CRC-25-0064)
Supplement: Supplementary Figure 7 — EVs from OVCAR3 do not induce detectable DNA damage relative to controls in short term exposure. [file crc-25-0064_supplementary_figure_7_suppsf7.docx]

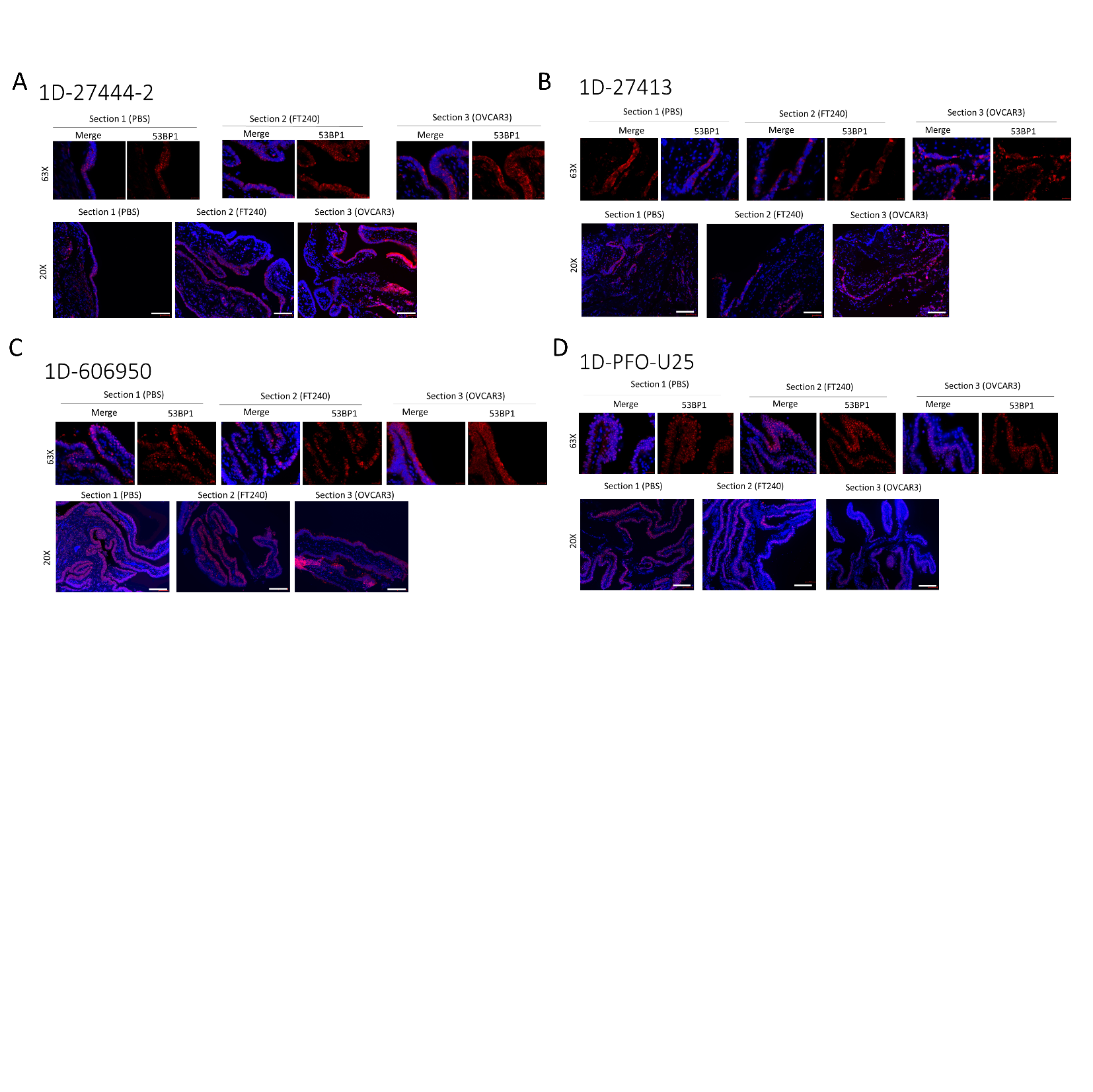


**Supplementary Figure 7. EVs from OVCAR3 do not induce detectable DNA damage relative to controls in short term exposure.**

Staining for nuclei (DAPI, blue) and DNA damage marker 53BP1 (red) in four different tissue samples following 1-day treatment with EVs**. A)** Tissue 27444, **B)** tissue 27431, **C)** tissue 606950, and **D)** tissue U25. No clear trend in DNA damage was observed. Scale bar = 100 µm.
