# Supplementary material for: Defining the Ovarian Cancer Precancerous Landscape through Modeling Fallopian Tube Epithelium Reprogramming Driven by Extracellular Vesicles
Source: Cancer Res Commun. 2025 Aug 4;5(8):1266–81. doi: 10.1158/2767-9764.CRC-25-0064 (PMC12319521; doi:10.1158/2767-9764.CRC-25-0064)
Supplement: Supplementary Figure 8 — EVs from OVCAR3 do not induce detectable DNA damage relative to controls in long term exposure. [file crc-25-0064_supplementary_figure_8_suppsf8.docx]

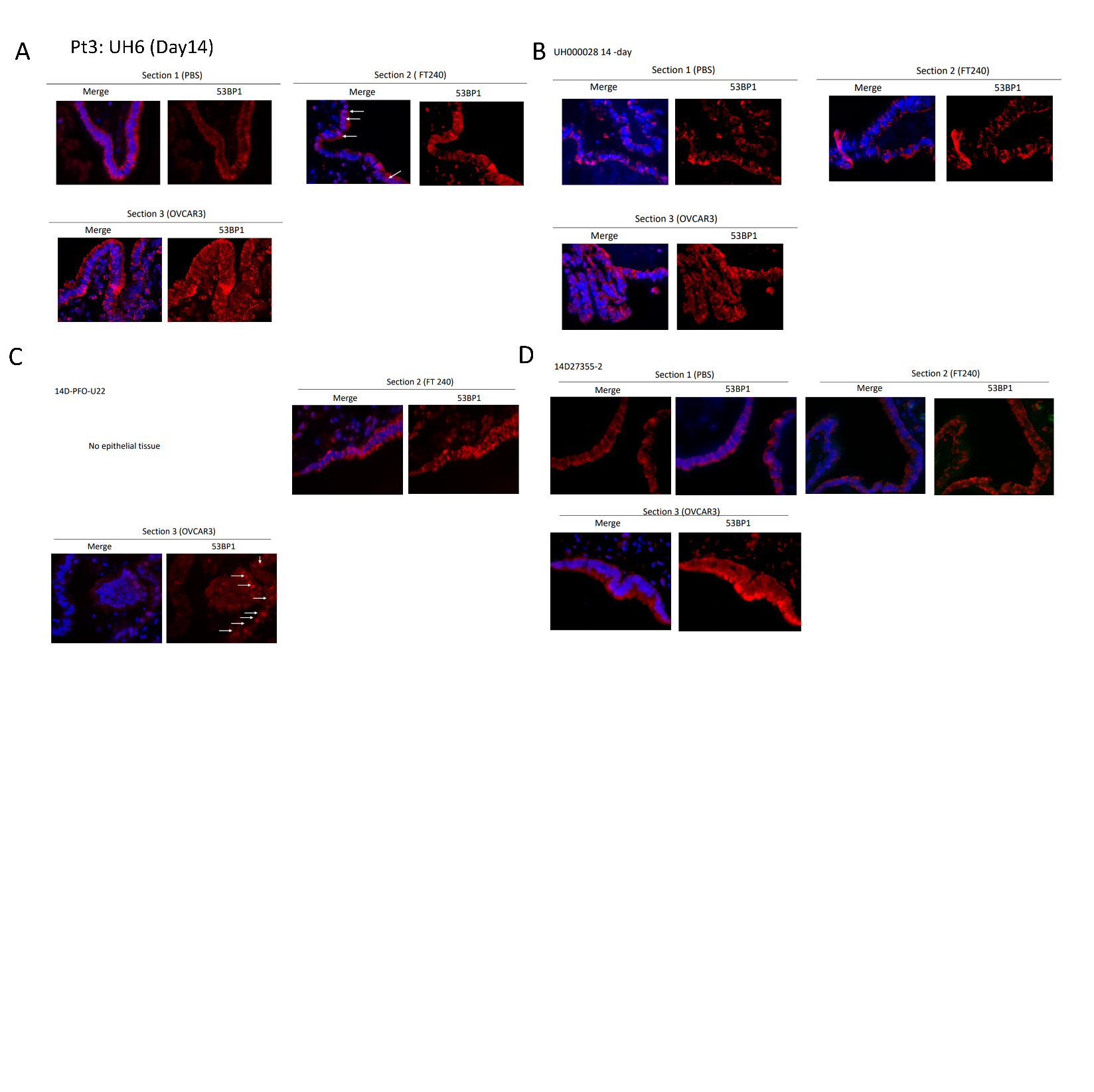


**Supplementary Figure 8. EVs from OVCAR3 do not induce detectable DNA damage relative to controls in long term exposure**.

Staining for nuclei (DAPI, blue) and the DNA damage marker 53BP1 (red) in four different tissue samples following 14-day treatment long term stimulation with EVs**. A)** Tissue UH6, **B)** tissue UH28, **C)** Tissue U22, and **D)** Tissue 27355. No clear trend in DNA damage was observed. Cells with high DNA damage are indicated by the white arrows.
