# Supplementary material for: Defining the Ovarian Cancer Precancerous Landscape through Modeling Fallopian Tube Epithelium Reprogramming Driven by Extracellular Vesicles
Source: Cancer Res Commun. 2025 Aug 4;5(8):1266–81. doi: 10.1158/2767-9764.CRC-25-0064 (PMC12319521; doi:10.1158/2767-9764.CRC-25-0064)
Supplement: Supplementary Figure 9 — PAX2 expression is downregulated by OVCAR3 EVs [file crc-25-0064_supplementary_figure_9_suppsf9.docx]

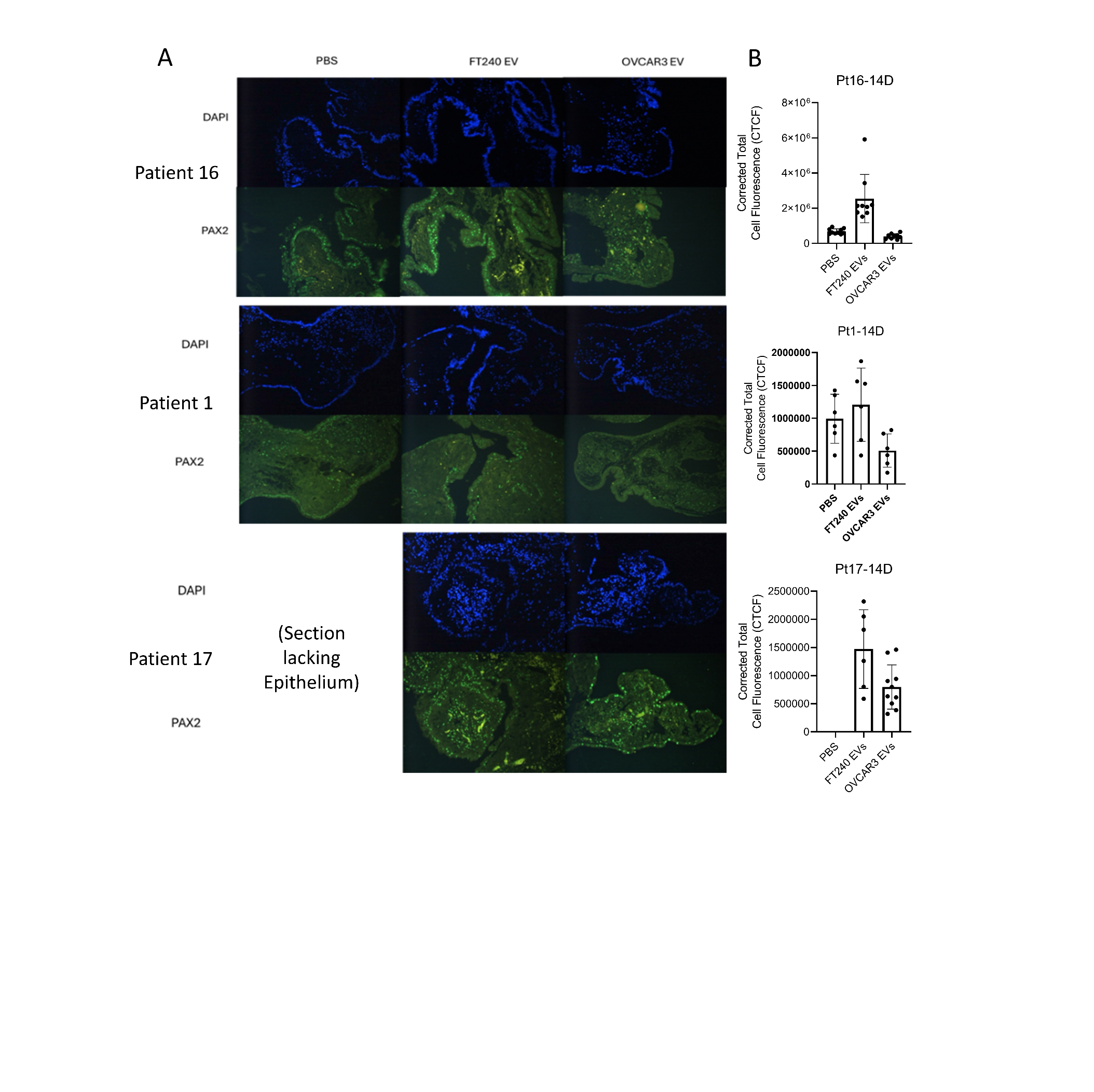


**Supplementary Figure 9. PAX2 expression is downregulated by OVCAR3 EVs**

**A)** Immunofluorescence staining of PAX2 in FT slides, comparing PBS (left), FT240 EV treated (middle), and OVCAR3 EV treated (right). Blue = DAPI, Green = PAX2; Top = Slides from Pt16, Middle = Pt1, Bottom = Pt17; **B)** Bar plots showing of PAX2 intensity in samples (dots represent regions of epithelium)
