# Supplementary material for: Defining the Ovarian Cancer Precancerous Landscape through Modeling Fallopian Tube Epithelium Reprogramming Driven by Extracellular Vesicles
Source: Cancer Res Commun. 2025 Aug 4;5(8):1266–81. doi: 10.1158/2767-9764.CRC-25-0064 (PMC12319521; doi:10.1158/2767-9764.CRC-25-0064)
Supplement: Supplementary Figure 11 — GO Biological pathways downregulated by FT240 EVs. [file crc-25-0064_supplementary_figure_11_suppsf11.docx]

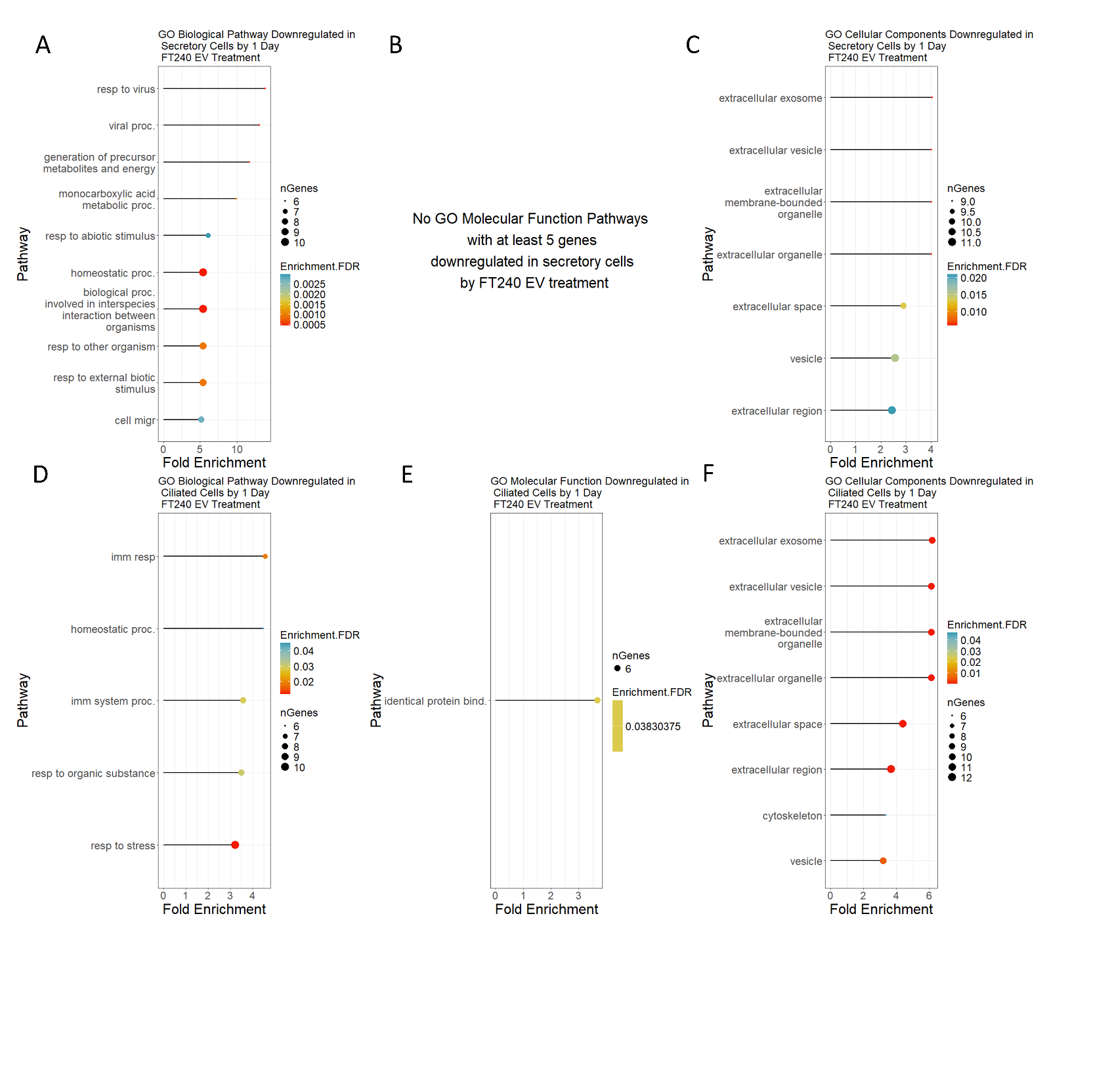


**Supplementary Figure 11. GO Biological pathways downregulated by FT240 EVs.**

**A-F)** GO analysis showing pathways downregulated by FT240 EVs in secretory **(A-C)** and ciliated **(D-F)** cells following one day of treatment.
