# Supplementary material for: Defining the Ovarian Cancer Precancerous Landscape through Modeling Fallopian Tube Epithelium Reprogramming Driven by Extracellular Vesicles
Source: Cancer Res Commun. 2025 Aug 4;5(8):1266–81. doi: 10.1158/2767-9764.CRC-25-0064 (PMC12319521; doi:10.1158/2767-9764.CRC-25-0064)
Supplement: Supplementary Figure 12 — KEGG and reactome pathways upregulated and downregulated by OVCAR3 EVs. [file crc-25-0064_supplementary_figure_12_suppsf12.docx]

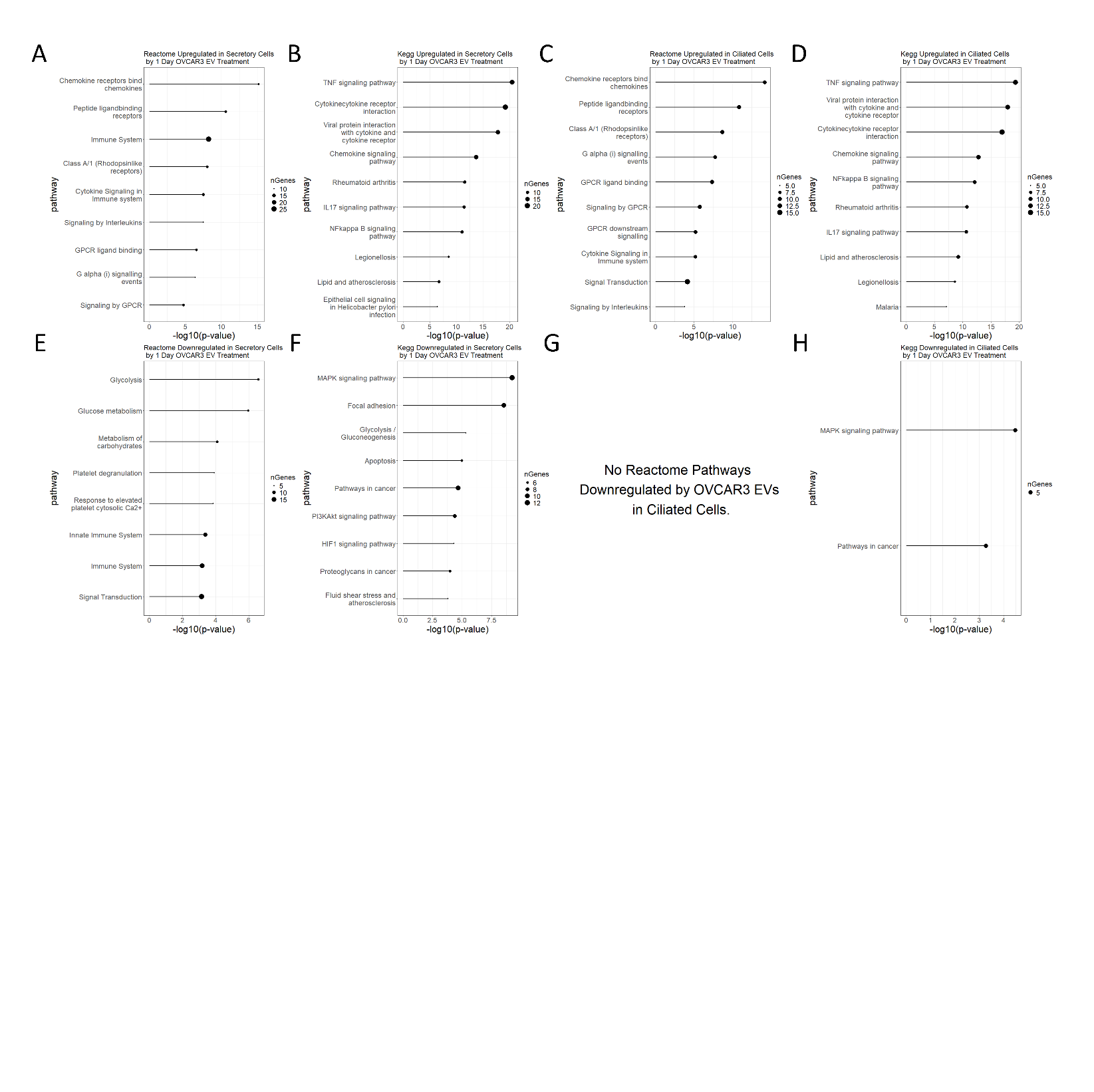


**Supplementary Figure 12. KEGG and reactome pathways upregulated and downregulated by OVCAR3 EVs.**

**A-H)** KEGG and reactome analysis showing pathways upregulated and downregulated by OVCAR3 EVs. **A)** Reactome analysis shown pathways upregulated in secretory cells by OVCAR3 EVs. **B)** KEGG pathway analysis showing pathways upregulated in secretory cells by OVCAR3 EVs. **C)** Reactome analysis showing pathways upregulated in ciliated cells by OVCAR3 EV treatment. **D)** KEGG pathway analysis showing pathways upregulated in ciliated cells by OVCAR3 EV treatment. **E)** Reactome pathway analysis showing pathways downregulated in secretory cells. **F)** KEGG pathway analysis showing pathways downregulated in secretory cells. **G)** Reactome pathway analysis showing pathways downregulated in ciliated cells. **H)** KEGG pathway analysis showing pathways downregulated in ciliated cells.
