# Supplementary material for: Defining the Ovarian Cancer Precancerous Landscape through Modeling Fallopian Tube Epithelium Reprogramming Driven by Extracellular Vesicles
Source: Cancer Res Commun. 2025 Aug 4;5(8):1266–81. doi: 10.1158/2767-9764.CRC-25-0064 (PMC12319521; doi:10.1158/2767-9764.CRC-25-0064)
Supplement: Supplementary Figure 14 — Unsupervised clustering of Figure 5E. [file crc-25-0064_supplementary_figure_14_suppsf14.docx]

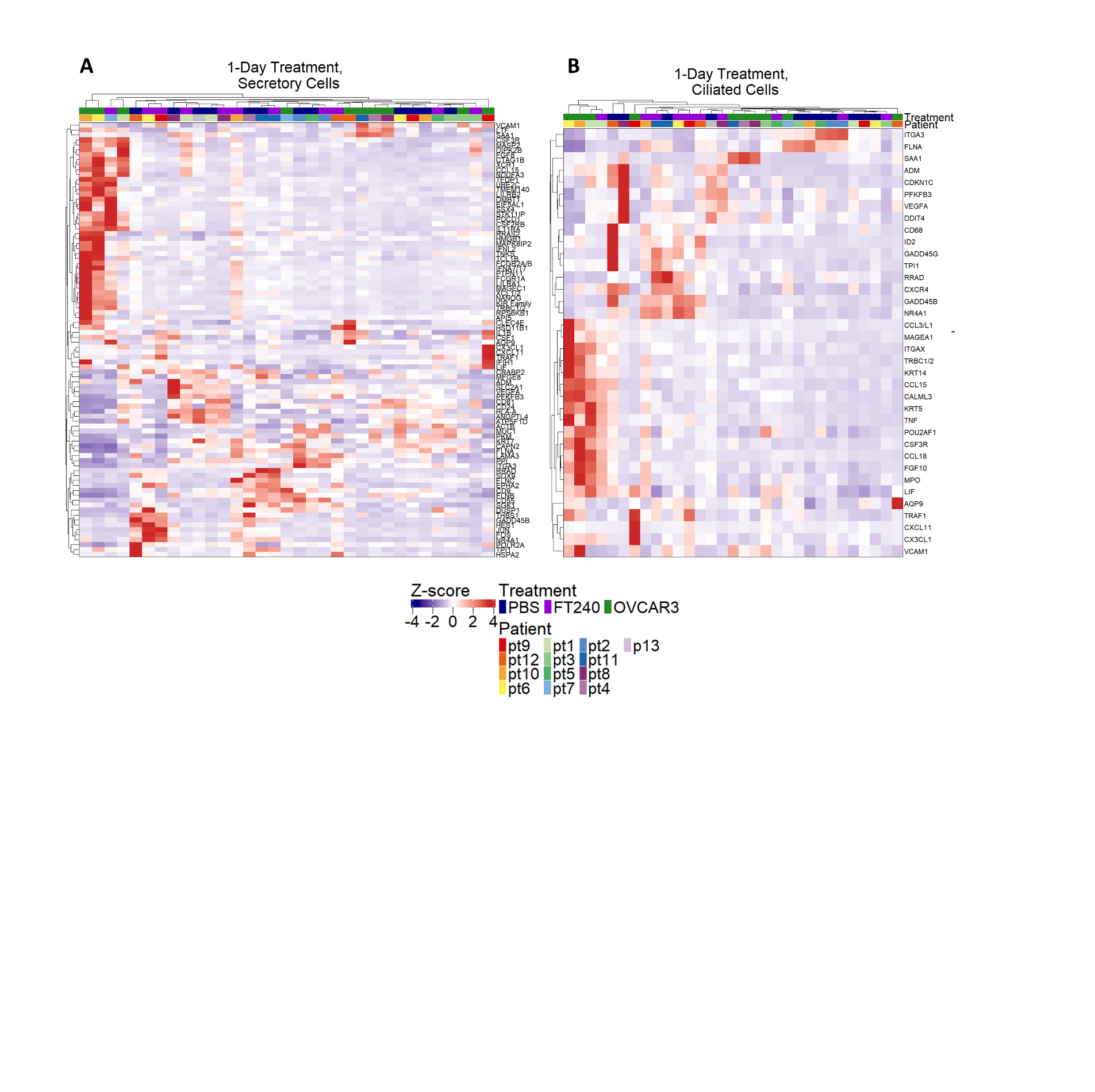


**Supplementary Figure 14. Unsupervised clustering of Figure 5E.**

**A)** Heatmap showing unsupervised clustering of Figure 5E, secretory cells. **B)** Heatmap showing unsupervised clustering, figure 5E, ciliated cells. Green = OVCAR3 EV treatment, Purple = FT240 EV treatment, Blue = PBS treatment.
