# Supplementary material for: Defining the Ovarian Cancer Precancerous Landscape through Modeling Fallopian Tube Epithelium Reprogramming Driven by Extracellular Vesicles
Source: Cancer Res Commun. 2025 Aug 4;5(8):1266–81. doi: 10.1158/2767-9764.CRC-25-0064 (PMC12319521; doi:10.1158/2767-9764.CRC-25-0064)
Supplement: Supplementary Figure 15 — Key IFN-γ pathway genes are upregulated by OVCAR3 EV treatment. [file crc-25-0064_supplementary_figure_15_suppsf15.docx]

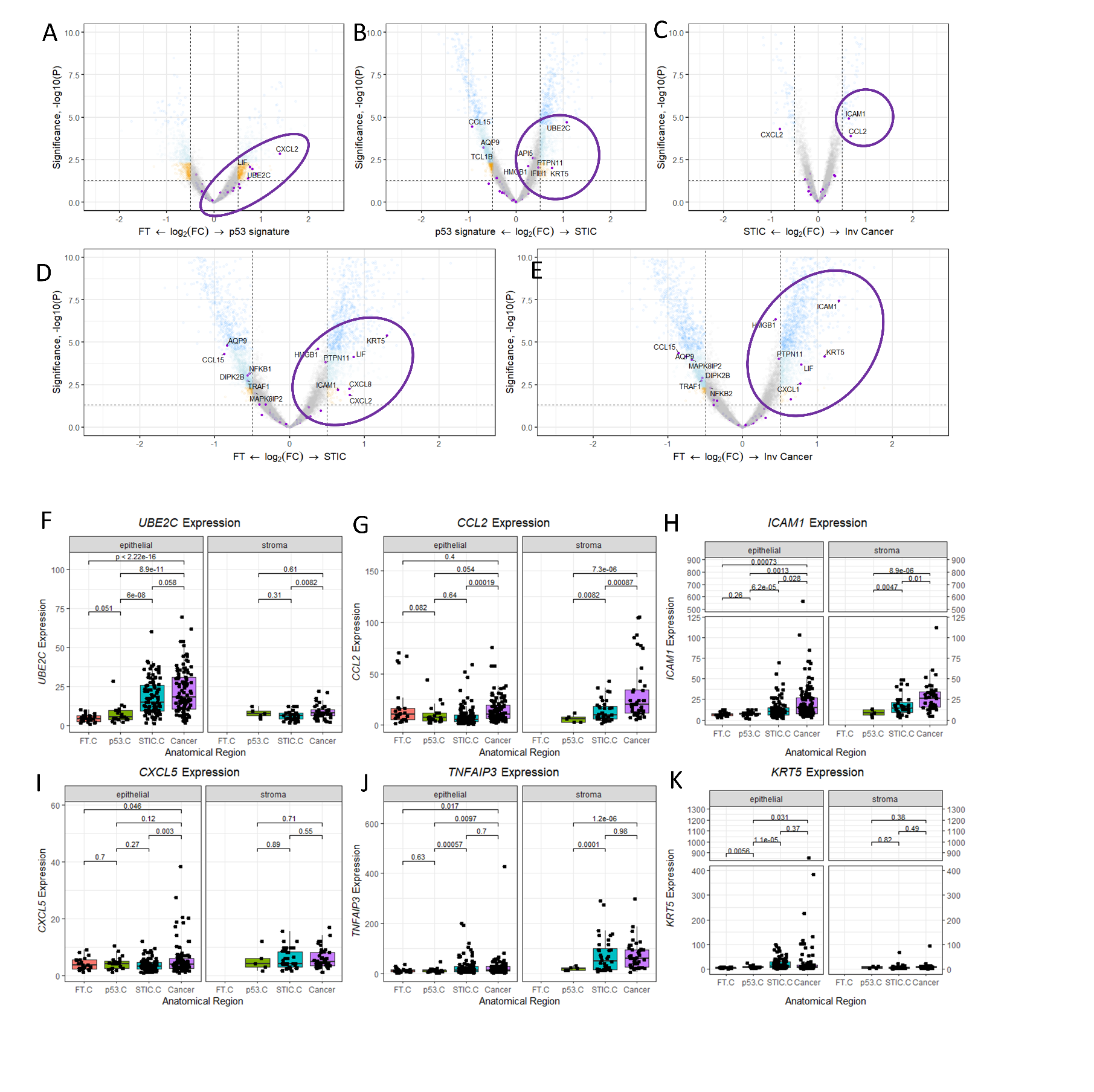


**Supplementary Figure 15. Key IFN-γ pathway genes are upregulated by OVCAR3 EV treatment.**

**A-D)** volcano plots comparing EV treatment conditions to PBS controls. A) OVCAR3 vs PBS treated tissue transcript expression in Secretory cells. **B)** FT240 v PBS in secretory cells. **C)** OVCAR3 v PBS in ciliated cells. **D)** FT240 v PBS in ciliated cells. Genes involved in the IFN-γ pathway are highlighted in purple. Gene list from (Kader et al. 2024). Genes in pathway upregulated by EV treatment circled.
