# Supplementary material for: Defining the Ovarian Cancer Precancerous Landscape through Modeling Fallopian Tube Epithelium Reprogramming Driven by Extracellular Vesicles
Source: Cancer Res Commun. 2025 Aug 4;5(8):1266–81. doi: 10.1158/2767-9764.CRC-25-0064 (PMC12319521; doi:10.1158/2767-9764.CRC-25-0064)
Supplement: Supplementary Figure 16 — Transcripts upregulated by OVCAR3 EVs are also upregulated in progression from precursor lesion to invasive cancer. [file crc-25-0064_supplementary_figure_16_suppsf16.docx]

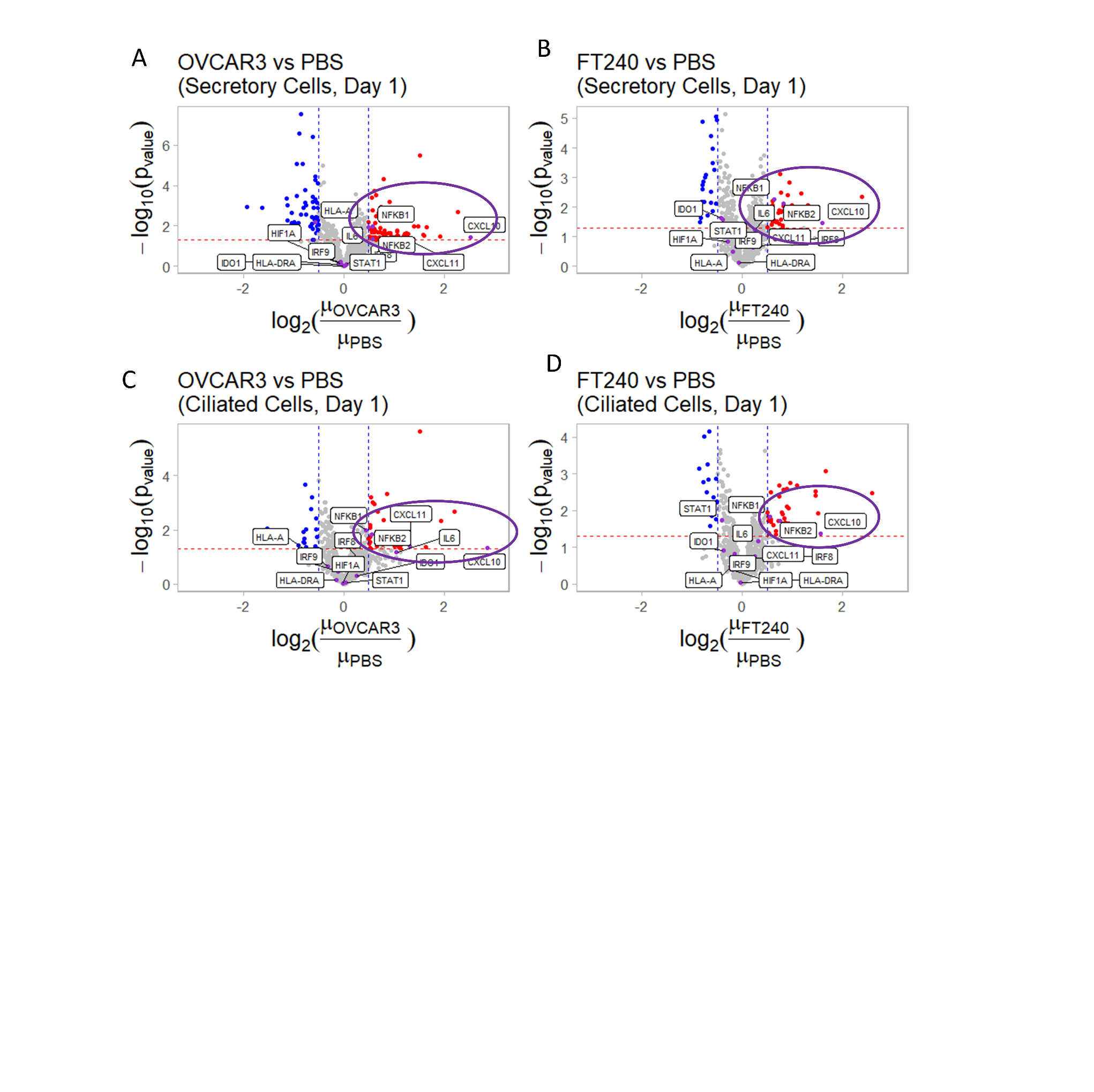


**Supplementary Figure 16. Transcripts upregulated by OVCAR3 EVs are also upregulated in progression from precursor lesion to invasive cancer.**

All transcript expression data from GEO: GSE281193 (Kader et al. 2024). **A-E)** Volcano plots showing comparisons between epithelial cells found in **A)** FT and p53 signature, **B)** p53 signature and STIC lesion, **C)** STIC lesion and invasive cancer, **D)** FT and STIC lesion, and **E)** FT and Invasive Cancer. Genes upregulated by OVCAR3 EVs in our dataset are highlighted in purple. Genes that are upregulated during progression circled. All lesions and FT samples come from cancer patients. **F-H)** Boxplots comparing transcript expression across precursor lesions for **F)** UBE2C, **G)** CCL2, **H)** ICAM1, **I)** CXCL5, **J)** TNFAIP3, and **K)** KRT5. P-value calculated using t-test. Stroma refers to stromal cells adjacent to epithelial samples. Following the original dataset (Kader et al. 2024), FT.C = Fallopian tube of cancer patient, p53.C = p53 signature in cancer patient, STIC.C = Serous tubal intraepithelial carcinoma in cancer patient.
