# Supplementary material for: Defining the Ovarian Cancer Precancerous Landscape through Modeling Fallopian Tube Epithelium Reprogramming Driven by Extracellular Vesicles
Source: Cancer Res Commun. 2025 Aug 4;5(8):1266–81. doi: 10.1158/2767-9764.CRC-25-0064 (PMC12319521; doi:10.1158/2767-9764.CRC-25-0064)
Supplement: Supplementary Methods [file crc-25-0064_supplementary_methods_suppsm.docx]

**SUPPLEMENTARY METHODS**

**Full Differential Ultracentrifugation Procedure**

Brief summary: Following removal of cell debris and apoptotic bodies, medium was spun at

10,000 x g to pellet large EVs, followed by two spins at 100,00 x g to pellet small EVs. During EV collection, cells were grown in medium supplemented with EV-depleted FBS to reduce FBS-derived EV contamination. Immediately after collection, conditioned medium was centrifuged at 500 x g for 10 min to remove cell debris, followed by a second spin at 2,000 x g for 20 min to remove apoptotic bodies. Medium was then frozen at -20 °C until a sufficient volume was collected for ultracentrifugation (~500 mL). To remove large EVs, conditioned medium was spun at 10,000 x g for 1 h15 min, after which the remaining medium was decanted into clean centrifuge tubes and spun at 100,000 x g for 1 h and 30 min to pellet small EVs. The supernatant of the second spin was discarded, followed by resuspension of the pellet in ~ 45 mL of filtered phosphate-buffered saline (PBS) and a third and final spin at 100,000 x g for 1 hand 30 min. The final supernatant was discarded and the pellet was resuspended in ~100-200 µL of filtered PBS, followed by storage at -80 °C until use. To avoid damage to the EV sample via repetitive freeze-thaw, the sample was separated into two aliquots of equal concentration before freezing, one of which was used for analysis, while the other was stored for EV treatment.

**TEM Imaging of EVs**

Purified sEVs were diluted in filtered PBS for TEM preparation, using freshly thawed samples for imaging. Carbon-film-coated 300-mesh copper grids treated with glow discharge were placed on 20 µL of the purified sEV solution for 20 min. The grids were subsequently rinsed with six sequential droplets of distilled water, followed by staining for 5 s with 1% uranyl acetate. The grids were air-dried for 15 min before imaging. All the TEM images were obtained using a JEOL JEM-1400 transmission electron microscope. All imaging was completed within two days of the initial staining.

**Mass Spectrophotometry Procedure for Cell-line EVs**

Label-free LC-MS/MS was performed according to a previously established protocol (1-3). The sEVs were lysed using SDS lysis buffer to release intravesicular proteins. The sEV proteins were processed to tryptic peptides using filter-aided sample preparation (4). Tryptic peptides were then separated by reverse-phase XSelect CSH C18 2.5 µm resin (Waters, Milford, MA, USA) on an in-line 150 × 0.075 mm column, using an UltiMate 3000 RSLCnano system (Thermo Fisher Scientific, Waltham, MA, USA). Peptides were eluted using a 90 min gradient from 98:2 to 65:35 buffer A:B ratio. Mobile phase A consisted of 0.1% formic acid in 0.5% acetonitrile, and mobile phase B consisted of 0.1% formic acid in 99.9% acetonitrile. An Orbitrap Eclipse Tribrid mass spectrometer (Thermo Fisher Scientific) was used for the mass spectrometric analysis. Peptides were ionized by electrospray (2.4 kV), and MS data were acquired using an FTMS analyzer in profile mode at a resolution of 120,000 over 375to 1200 m/z. Following HCD activation, MS/MS data were acquired using an ion trap analyzer in centroid mode and a normal mass range, with a normalized collision energy of 30%. MaxQuant Version 2.0.3.1 (Max Planck Institute of Biochemistry, Martinsried, Germany) was used for protein identification, with a parent ion tolerance of 2.5 ppm and a fragment ion tolerance of 0.5 Da. Scaffold Q+S Version 5.1.2 (Proteome Software, Portland, Oregon, USA) was used to verify MS/MS-based peptide and protein identifications.

**Immunohistochemistry Full Procedure**

The fallopian tube epithelium was fixed in 4% paraformaldehyde following the 24-hour or 14-day treatment with OVCAR3-derived sEVs on PREDICT-MOS. Paraffin-embedded slides were deparaffinized using two xylene washes, each for 10 min, followed by rehydration in an ethanol gradient of 100%, 95%, 70%, 50%, and ddH_2_O, for 3 min each. Antigen retrieval was performed in 0.1 M Na citrate (pH 6) in a microwave set on high for 2 min, followed by 13 min on low (power 10), after which the slides were permitted to cool for 30 min. Slides were blocked with avidin and biotin, and then with 10% goat serum in 3% BSA-TBS solution for 1 h. Primary antibodies against CCL2 (Novus Cat# NBP1-07035SS, RRID:AB_1625611), VCAM1 (Thermo Fisher Scientific Cat# MA5-31965, RRID:AB_2809259), FLNA (Proteintech Cat# 67133-1-Ig, RRID:AB_2882432), TPI1 (Proteintech Cat# 10713-1-AP, RRID:AB_2207716), and TXNIP (Thermo Fisher Scientific Cat# 40-3700, RRID:AB_2533462) were incubated overnight at 4 °C. The slides were washed in 0.1% TBS-T thrice, each for 5 min, and incubated with goat secondary antibodies conjugated to biotin in 3% BSA-TBS for 30 min the following day. After repeating the 0.1% TBS-T wash thrice, the slides were incubated in ABC reagent for 30 min. DAB substrate was added to the slides in the presence of HRP and developed for 3–10 min. The reaction was quenched with ddH2O, and the tissues were counterstained with hematoxylin.

**PAX2 Immunofluorescence Full Procedure**

Slides were deparaffinized in xylene and rehydrated in a gradient of ethanol. The slides were then heated in 0.01M sodium citrate for antigen retrieval. Tissue slides were permeabilized with 0.2% Triton in PBS for 20 mins at room temperature, followed by blocking with 1% BSA in PBS. The slides were washed three times with 0.05% PBS-T. The primary antibody to PAX2 (Abcam Cat# ab79389, RRID:AB_1603338) was added to the slides and incubated overnight at 4 °C. The next day, the slides were washed three times with 0.05% PBS-T and were incubated with the anti-rabbit fluorescent secondary antibody with Alexa 488 (Thermo Fisher Scientific Cat# A-11034, RRID:AB_2576217) for 1 hour at room temperature in the dark. Slides were then washed three times in 0.05% PBS-T and incubated with DAPI (Thermo-Fisher, EN62248) for 10 mins. The slides were then washed again with PBST for 2 times and mounted with VECTASHIELD® Antifade Mounting Medium with DAPI (VectorLabs, H-1200-10).

**Mass Spectrophotometry Procedure for hFTE-derived EVs**

EV protein from each sample was reduced, alkylated, and digested using a single-pot, solid-phase-enhanced sample preparation (5) with sequencing-grade modified porcine trypsin (Promega). Tryptic peptides were trapped and eluted on a 3.5 µm CSH C18 resin (Waters) (4 mm x 75 µm) and then separated by reverse-phase XSelect CSH C18 2.5 um resin (Waters) on an in-line 150 x 0.075 mm column using an UltiMate 3000 RSLCnano system (Thermo Fisher Scientific). Peptides were eluted at a flow rate of 0.300 µL/min using a 60 min gradient from 98% Buffer A:2% Buffer B to 95:5 at 2.0 minutes to 80:20 at 39.0 minutes to 60:40 at 48.0 minutes to 10:90 at 49.0 minutes and hold until 53.0 minutes and then equilibrated back to 98:2 at 53.1 minutes until 60 minutes. The eluted peptides were ionized by electrospray (2.4 kV) through a heated capillary (275 °C), followed by data collection on an Orbitrap Exploris 480 mass spectrometer (Thermo Fisher Scientific).

Precursor spectra were acquired with a scan from 385-1015 Th at a resolution of 60,000 with 100% AGC, maximum time of 50 ms, and an RF parameter of 40%. The DIA was configured on the Orbitrap 480 to acquire 50 x 12 Th isolation windows at 15,000 resolution, normalized AGC target 500%, and maximum injection time 40 ms). A second DIA was acquired in a staggered window (12 Th) pattern with optimized window placements. Following data acquisition, data were searched using Spectronaut (Biognosys version 18.3) against the UniProt Homo sapiens (March 2023) using the directDIA method with an identification precursor and protein q-value cutoff of 1%, generate decoys set to true, the protein inference workflow set to max LFQ, inference algorithm set to IDPicker, quantity level set to MS2, cross-run normalization set to false, and the protein grouping quantification set to median peptide and precursor quantity. Protein MS2 intensity values were assessed for quality using ProteiNorm (6) and the data was normalized using Robust Linear Regression (RLR).

Citations

1. Zha D, Rayamajhi S, Sipes J, Russo A, Pathak HB, Li K*, et al.* Proteomic Profiling of Fallopian Tube-Derived Extracellular Vesicles Using a Microfluidic Tissue-on-Chip System. Bioengineering (Basel) **2023**;10

2. Atay S, Wilkey DW, Milhem M, Merchant M, Godwin AK. Insights into the Proteome of Gastrointestinal Stromal Tumors-Derived Exosomes Reveals New Potential Diagnostic Biomarkers*. Molecular & Cellular Proteomics **2018**;17:495-515

3. Trinidad CV, Pathak HB, Cheng S, Tzeng SC, Madan R, Sardiu ME*, et al.* Lineage specific extracellular vesicle-associated protein biomarkers for the early detection of high grade serous ovarian cancer. Sci Rep **2023**;13:18341

4. Wiśniewski JR, Zougman A, Nagaraj N, Mann M. Universal sample preparation method for proteome analysis. Nat Methods **2009**;6:359-62

5. Hughes CS, Moggridge S, Müller T, Sorensen PH, Morin GB, Krijgsveld J. Single-pot, solid-phase-enhanced sample preparation for proteomics experiments. Nat Protoc **2019**;14:68-85

6. Graw S, Tang J, Zafar MK, Byrd AK, Bolden C, Peterson EC*, et al.* proteiNorm - A User-Friendly Tool for Normalization and Analysis of TMT and Label-Free Protein Quantification. ACS Omega **2020**;5:25625-33
