# Supplementary figures and images for: Defining the Ovarian Cancer Precancerous Landscape through Modeling Fallopian Tube Epithelium Reprogramming Driven by Extracellular Vesicles
Source: Cancer Res Commun. 2025 Aug 4;5(8):1266–81. doi: 10.1158/2767-9764.CRC-25-0064 (PMC12319521; doi:10.1158/2767-9764.CRC-25-0064)

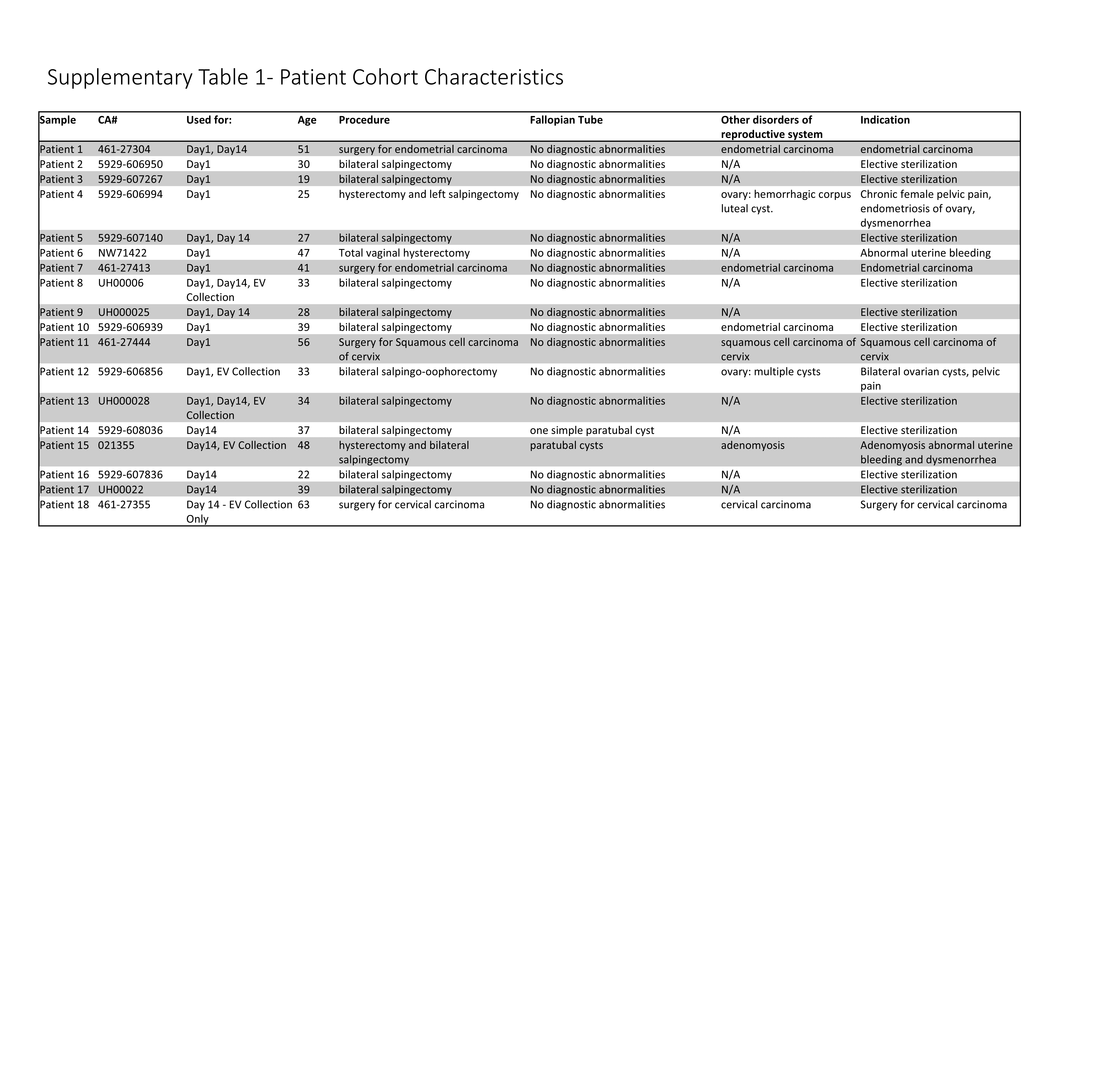

Supplement: Supplementary Table 1 — Patient Cohort Characteristics [file crc-25-0064_supplementary_table_1_suppst1.docx]

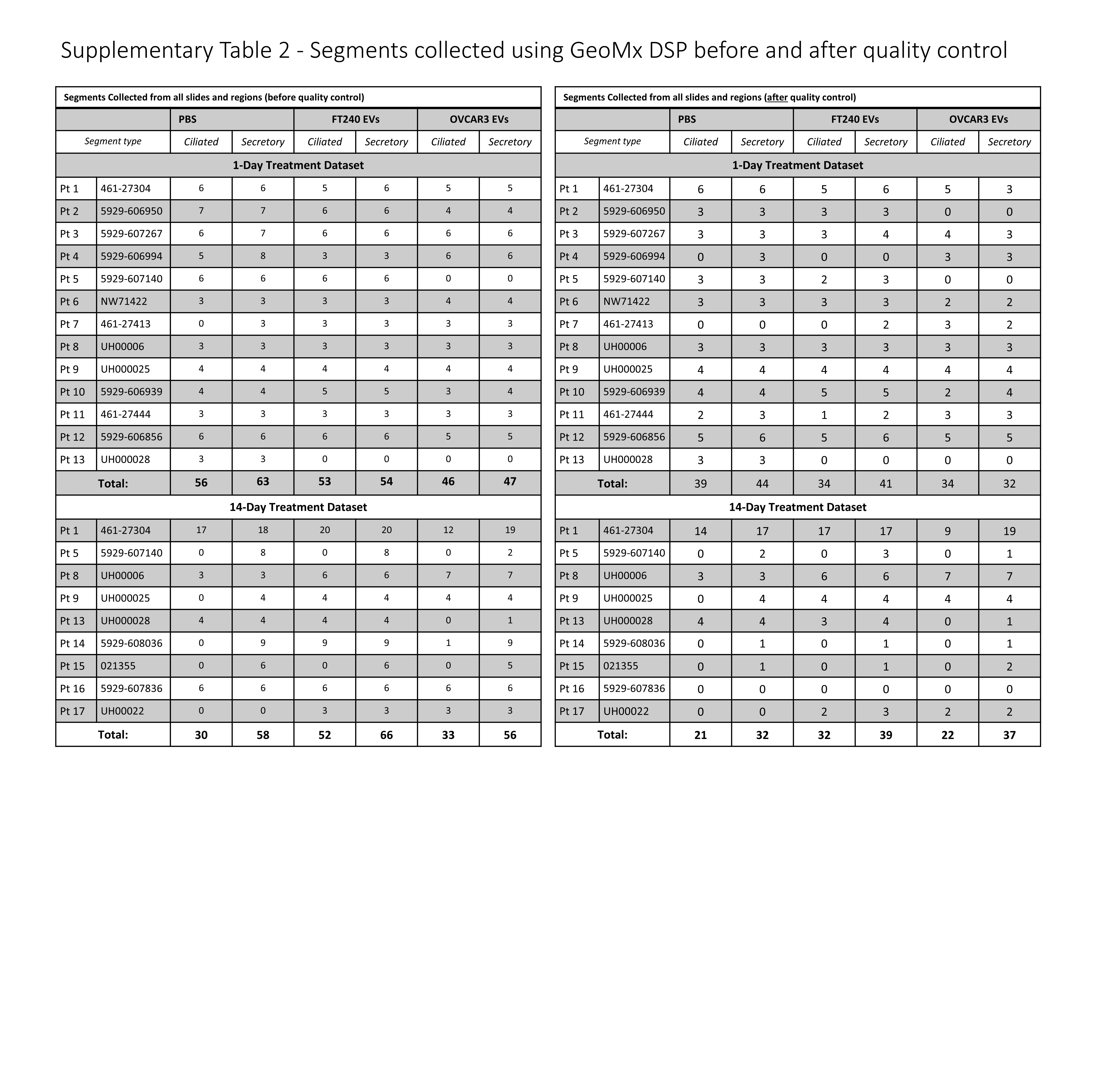

Supplement: Supplementary Table 2 — Segments collected using GeoMx DSP before and after quality control [file crc-25-0064_supplementary_table_2_suppst2.docx]
